# Genome Sequence of Peacock Reveals the Peculiar Case of a Glittering Bird

**DOI:** 10.3389/fgene.2018.00392

**Published:** 2018-09-19

**Authors:** Shubham K. Jaiswal, Ankit Gupta, Rituja Saxena, Vishnu P. K. Prasoodanan, Ashok K. Sharma, Parul Mittal, Ankita Roy, Aaron B. A. Shafer, Nagarjun Vijay, Vineet K. Sharma

**Affiliations:** ^1^Metagenomics and Systems Biology Group, Department of Biological Sciences, Indian Institute of Science Education and Research Bhopal, Bhopal, India; ^2^Forensic Science and Environmental and Life Sciences, Trent University, Peterborough, ON, Canada; ^3^Computational Evolutionary Genomics Lab, Department of Biological Sciences, Indian Institute of Science Education and Research Bhopal, Bhopal, India

**Keywords:** peacock genome, peafowl, comparative genomics, dN/dS, positive selection, adaptive evolution, Hamilton–Zuk hypothesis

## Abstract

The unique ornamental features and extreme sexual traits of Peacock have always intrigued scientists and naturalists for centuries. However, the genomic basis of these phenotypes are yet unknown. Here, we report the first genome sequence and comparative analysis of peacock with the high quality genomes of chicken, turkey, duck, flycatcher and zebra finch. Genes involved in early developmental pathways including TGF-β, BMP, and Wnt signaling, which have been shown to be involved in feather patterning, bone morphogenesis, and skeletal muscle development, revealed signs of adaptive evolution and provided useful clues on the phenotypes of peacock. Innate and adaptive immune genes involved in complement system and T-cell response also showed signs of adaptive evolution in peacock suggesting their possible role in building a robust immune system which is consistent with the predictions of the Hamilton–Zuk hypothesis. This study provides novel genomic and evolutionary insights into the molecular understanding toward the phenotypic evolution of Indian peacock.

## Introduction

Among the most conspicuous species of bird is the Indian peafowl (*Pavo cristatus*), which once puzzled the greatest naturalist, Charles Darwin, who wrote – “the sight of a feather in a Peacock’s tail, whenever I gaze at it, makes me sick” (Darwin, 1887). The presence of an exceptional ornamental plumage with large tail-coverts in peacock, which makes it vulnerable to predators attack, posed a question on his theory of natural selection. Darwin’s problem was to explain the presence of peacock’s train through his theory of natural selection as it seemed to be disadvantageous to male peafowl. Subsequently, Charles Darwin justified the presence of such ornamental traits including peacock’s train by proposing the theory of sexual selection according to which these ornamental characters provide advantage to an individual over others of same species by providing higher reproductive success (Darwin, 1888).

The *Pavo* genus from the family *Phasianidae* has two known species, *P. cristatus* (Blue peafowl) and *Pavo muticus* (Green peafowl), which diverged about 3 million years ago (Ouyang et al., 2009). The Blue peafowl (Indian Peacock) is endemic to the Indian subcontinent, whereas, the Green Peafowl is found across Southeast Asia. Peacock (male peafowl) is one of the largest known bird among pheasants and flying birds. It shows sexual dimorphism, polygamy with no paternal care to offspring, and an elaborate male display during courtship (Zahavi, 1975; Ramesh and Mcgowan, 2009). Sexual selection is considered extreme in peacock, which is dependent upon both the ornamental display (glittering train and crest plumage) and behavioral traits (Loyau et al., 2005b). These ornamental features are also used as an honest signal about their immunocompetence to the peahen, which helps in the selection of individuals with better immunity (Loyau et al., 2005a). Though, the male traits are testosterone-dependent in peacock, the large train is the default state since the peahen also shows the development of this train after ovariectomy (Owens and Short, 1995).

The existence of intricate ornaments in peacock has perplexed scientists for decades and has led to several ecological and population-based studies (Zahavi, 1975; Loyau et al., 2005b; Ramesh and Mcgowan, 2009). However, the genomic details about the phenotypic evolution of this species are still unknown. Therefore, we carried out a comprehensive comparative genomics analysis of *P. cristatus* (Blue Peafowl) to decipher the genomic evolution of this species. The ornamental and sexual characteristics of peacock are distinct from other birds and are absent in closely related species such as chicken and turkey; this presents an ideal set-up to look for the genomic changes underlying the phenotypic divergence of peacock. Moreover, the origin of ocelli in the peacock feathers is relatively recent and independent of the other ocellated genera of birds such as *Argusianus* and *Ploypectron* in the *Phasianidae* family (Sun et al., 2014). Thus, studying the molecular evolution of peacock at genome-wide level is crucial to have a holistic understanding about these phenotypes. We also carried out a comprehensive comparative genome-wide analysis of the peacock genome (order *Galliformes*) with the high quality genomes of five other birds under the class Aves: chicken and turkey (order *Galliformes*), duck (order *Anseriformes*), and flycatcher and zebra finch (order *Passeriformes*). The comparative genome-wide analysis of peacock with five other related birds provided novel genomic insights into peacock genome evolution.

## Materials and Methods

### Sample Collection, DNA Isolation, and Sequencing of Peacock Genome

Approximately 2 ml blood was drawn from the medial metatarsal vein of a 2 years old Indian peacock at Van Vihar National Park, Bhopal, India and was collected in EDTA-coated vials. This study protocol was carried out in accordance with the approval of Institute Ethics committee of IISER Bhopal. The permission to collect blood samples was obtained from Principal Chief Conservator of Forests Bhopal. The fresh blood sample was immediately brought to the laboratory at 4°C and genomic DNA was extracted using DNeasy Blood and Tissue Kit (Qiagen, United States) following the manufacturer’s protocol. Sex of the bird was determined to be male by morphological identification and was confirmed using molecular sexing assay (**Supplementary Text S1**). Multiple shotgun genomic libraries were prepared using Illumina TruSeq DNA PCR-free library preparation kit and Nextera XT sample preparation kit (Illumina Inc., United States), as per the manufacturers protocol. The insert size for the TruSeq libraries was selected to be 550 bp and the average insert size for Nextera XT libraries was 300–1,200 bp (average 650). The sequencing library size for both the libraries was assessed on 2100 Bioanalyzer using High Sensitivity DNA kit (Agilent, United States). The libraries were quantified using KAPA SYBR FAST qPCR Master mix with Illumina standards and primer premix (KAPA Biosystems, Wilmington, MA, United States), and Qubit dsDNA HS kit on a Qubit 2.0 fluorometer (Life Technologies, United States) as per the Illumina suggested protocol. The normalized libraries were loaded on Illumina NextSeq 500 platform using NextSeq 500/550 v2 sequencing reagent kit (Illumina Inc., United States) and 150 bp paired-end sequencing was performed for all the libraries.

### Genome Assembly, Draft Genome Construction, and Genome Annotation

The preprocessing of raw reads was performed, which included filtering, normalization, and error correction (**Supplementary Text S2**). KmerGenie (v 1.7016) (Chikhi and Medvedev, 2013) was used with default parameters to determine the best k-mer size for *de novo* assembly. Using the high quality reads, contigs were assembled using ABySS (v2.0.2) (Jackman et al., 2017), with a k-mer size of 101. Gapcloser tool (v1.12) of SOAPdenovo (Luo et al., 2012) was used to close the gaps in the assembled genome. Finally, Agouti (v0.3.3) (Zhang et al., 2016) was used to further improve the genome assembly using the peacock transcriptome data available from a previous study (Harrison et al., 2015). The resultant assembly was validated using BUSCO scores (Simão et al., 2015). BUSCO has defined single-copy gene sets for major clades and provides quantitative measures for the assessment of genome assembly.

Additionally, a reference-based draft genome assembly was also performed by mapping the peacock sequencing reads to the chicken genome using BWA (v0.5.9) (Li and Durbin, 2009). The reads that aligned to the reference genome were sorted by the start position of their alignment to the reference genome, and a consensus sequence for all the chromosomes was built using SAMtools (v 0.1.19) (Li et al., 2009). The methods for genome annotation including repeat identification, predictions of tRNA, snoRNA, and microRNA are mentioned in **Supplementary Text S3**.

### Estimation of Effective Population Size (N_e_) History

The demographic history of the peacock was reconstructed by estimating the effective population size (N_e_) over time using Pairwise Sequentially Markovian Coalescent (PSMC) (Li and Durbin, 2011). The autosomal data of the peacock diploid genome sequence was filtered by excluding sites at which the inferred consensus quality was below 20, and the read depth was either one-third or more than twice of the average read depth across the genome. Since, mean coverage and percentage of missing data, both are important filtering thresholds in PSMC analysis, the minimum length of the contigs selected for carrying out the analysis was 5,000 bp based on no more than 25% of the missing data as suggested by Nadachowska-Brzyska et al. (2016) (**Supplementary Figure S1**). The resultant filtered genome sequence used for the analysis was 76% of the total genome. The parameters for PSMC were set to “N30 -t5 -r5 -p4+30^∗^24+610,” which were used previously for the 38 bird species (Nadachowska-Brzyska et al., 2015). Generation time and mutation rate are necessary to scale the results of PSMC analysis to real time. Hence, a generation time of 4 years was used in this analysis and was calculated as twice of the sexual maturity, which is 2 years for peacock according to “AnAge” database (Nadachowska-Brzyska et al., 2015). The mutation rate of 1.33e-09 was used as calculated in a previous study (Wright et al., 2015). It is known that the estimates of N_e_ from PSMC can be influenced by the quality of the genome and sequencing coverage. To ensure that our results are not strongly influenced by such artifacts, 100 bootstrap runs were performed to estimate the N_e_ from different parts of the genome to ascertain variability in the estimates of N_e_.

### Construction and Filtration of Gene-Set

The gene-set for the peacock was constructed using the combined approach of *ab initio* gene predictions and homology-based gene predictions. AUGUSTUS tool was utilized for *ab initio* gene predictions (Stanke et al., 2004). For the homology-based gene predictions mapping-based approach was carried out using the LASTZ tool (Harris, 2007). The detailed methods are provided in “Construction of gene-set” section of **Supplementary Text S3**. The final peacock gene-set using the combination of homology and *de novo* based approach comprised of 24,831 transcripts corresponding to 15,970 genes. All these gene sequences were translated and checked for premature stop codon. It was observed that 187 coding gene sequences corresponding to 136 genes had a premature stop codon in their sequence. Out of these, 130 genes had at least one valid coding transcript and were included in the analysis performed in this study.

### Identification of Orthologs

Reciprocal-best-BLAST-Hits (RBH) is well-known, and is among the preferred methods used for the large-scale comparative genomics (Wall et al., 2003). Using this method, a protein-coding sequence from a genome is considered orthologous to another protein-coding sequence present in a different genome if they appear as the best hits of each other in the pair-wise genome-wide homology search. Thus, the orthologs for peacock coding gene sequences were identified in the other bird genomes using the RBH approach through BLASTN (Altschul et al., 1997). Using this approach, the pair-wise orthologs were derived between peacock and each of the other bird species, and an intersection across all the pairs was taken to construct the combined orthologs across the six species.

### Sequence Alignment and Phylogenetic Tree Construction

All sequence alignments (DNA and Protein) used for the phylogenetic tree reconstruction and other sequence divergence analysis were generated using MUSCLE release 3.8.31 (Edgar, 2004). The phylogenetic tree analysis was carried out to determine the phylogeny from the concatenated alignments of orthologs including peacock, and to compare this phylogeny with other known phylogenies derived from different types of data. The likelihood-based tree-searching algorithm was used for phylogenetic tree reconstruction using PhyML version 3.1 (Guindon et al., 2010). In the case of nucleotide sequences, the default HKY85 model was used to construct the phylogenies of individual genes and mitochondrial genome, whereas the optimized GTR model was used to construct the phylogeny of concatenated alignments of orthologous genes. JTT model was utilized for constructing the phylogeny of protein sequences. The RY-coded phylogeny was constructed using PhyML version 3.1 with the default model of HKY85, whereas the Binary-coded phylogeny was constructed using RAxML version 8.2.10 (Stamatakis, 2014) with the default model “BINGAMMA” for binary data. To test for the robustness of the constructed phylogenetic trees, bootstrap technique was employed. A bootstrap value of 100 was used for individual gene based trees, RY-coded tree, and binary-coded tree. A bootstrap value of 1,000 was used for the mitochondrial and concatenated alignment-based trees (Hillis and Bull, 1993; Dunn et al., 2008). Time for the horizontal axis of the phylogenetic trees were derived from the TimeTree database (Hedges et al., 2006). The Robinson and Foulds distance (Robinson and Foulds, 1981) between individual gene trees and the concatenated alignment trees was calculated using the “RF.dist” function in R package “phangorn v2.0.3” (Schliep, 2010).

### Gene Gain/Loss Analysis

To estimate the gene gain and loss in gene families, CAFE (v3.1) (Han et al., 2013) with a random birth and death model was used. The species tree was constructed using NCBI taxonomy, and the branch lengths were used from TimeTree as described in Ensembl Compara pipeline (Vilella et al., 2009). The selected phylogenetic tree had the green anole as an out-group. Simulated data based on the properties of observed data was generated using the “genfamily” function and the significance of two-lambda model (separate lambda values for Galloanserae) was assessed against a global lambda model. The two-parameter model was found to fit the data better, as the observed likelihood ratio (LR) value calculated using the formula “LR = 2^∗^(score of global lambda model – score of multi-lambda model)” was greater than 95% of the distribution of simulated LRs. Further, CAFE analysis with random birth and death model was performed using two-parameter model.

### Identification of Genes With Multiple Signs of Adaptive Evolution

All validated peacock coding gene sequences (CDS) with >90% valid bases were analyzed through multiple sequence-based analysis such as dN/dS or ω (ratio of the rate of non-synonymous to the rate of synonymous substitutions) estimation, positive selection, and unique substitution to assess the adaptive sequence divergence. The functional analysis was performed using KEGG (Kanehisa and Goto, 2000), eggNOGs (Huerta-Cepas et al., 2016), and NCBI NR (O’leary et al., 2016) databases. The GO enrichment analysis was performed using a web-based tool “WebGestalt” (Zhang B. et al., 2005). Furthermore, the functional impact of the identified unique substitutions and other sequence variations were evaluated using SIFT (Sorting Intolerant from tolerant) analysis (Kumar et al., 2009). SIFT is a homology-based method, where the specific-amino acids of a protein sequence conserved across multiple species are considered to be functionally crucial.

#### Pair-Wise dN/dS Estimation

Based on the dN/dS or ω values, the positively selected (ω > 1), negatively selected (ω < 1), and neutrally selected (ω = 1) genes were identified. The dN/dS values for the peacock CDS were calculated using CODEML program of the PAML package 4.9 (Yang, 2007). The pairwise dN/dS analysis was performed on the pair-wise orthologous genes for seven different pairs: peacock-chicken, peacock-turkey, peacock-duck, peacock-zebra finch, peacock-flycatcher, chicken-turkey, and zebra finch-flycatcher using default parameters. To check for the convergence of calculated values, the iterations were performed with three different initial or fixed ω values, i.e., 0.5, 1, and 1.5, and only the coding gene sequences with consensus values were considered. To reduce the false positives and aberrant dN/dS values, the genes with dN/dS values >5 and between 1 and 1.2 (may indicate relaxation of constraint) were not used for the function interpretation of results.

#### Phylogenetically Corrected dN/dS Estimation

This analysis was only performed on the concatenated alignments of orthologs using CODEML program of the PAML package version 4.9 (Yang, 2007). The branch-specific model was utilized for the identification of branch-specific dN/dS values. In this model, each branch can have different dN/dS values. The maximum likelihood phylogenetic tree derived from the concatenated alignments of orthologs was used for the phylogenetic correction of branch-specific dN/dS value calculations.

#### Positive Selection Analysis

The multiple sequence alignment for each peacock coding gene sequence and the corresponding orthologs identified using RBH approach in the other five bird genomes were carried out using EMBOSS tranalign program (Rice et al., 2000). Furthermore, the Maximum Likelihood-based (ML) phylogenetic tree was constructed using the amino acid sequence of these orthologs. Based on the alignment and the phylogenetic tree, the calculations of likelihood scores with revised branch-site model A was performed to identify the signatures of positive selection in peacock for the considered coding gene sequence. This model tries to detect positive selection acting on specific sites on the particular specified branches (foreground branch) (Yang et al., 2005; Zhang J. et al., 2005). The “foreground branch” consisted of peacock, and the other branches constituted the “background branch.” The codons were categorized into previously assumed four classes in the model based on the foreground and background estimates of dN/dS (ω) values. The alternative hypothesis, according to which the foreground branch show positive selection with ω > 1 was compared with the null hypothesis, according to which all branches have the same ω = 1 value. The comparison was performed using Likelihood Ratio Test (LRT) values based chi-square test. The genes with FDR q-value (after multiple testing correction of LRT *p*-values) less than 0.1 were considered to be positively selected in peacock. Additionally, the amino acid sites under positive selection were identified using the Bayesian Empirical Bayes values for the branch-site model A (Zhang J. et al., 2005). This positive selection analysis was performed using CODEML program of the PAML package version 4.9 (Yang, 2007).

#### Unique Substitution Analysis

The peacock coding gene sequence and its orthologs identified from the five bird genomes were translated using EMBOSS transeq, and the protein sequence alignments were performed using MUSCLE release 3.8.31 (Edgar, 2004). Using custom-made Perl scripts, the positions at which the peacock protein showed amino acid substitutions in comparison to all the other five bird genomes were identified and reported as the unique substitutions in peacock genome. The functional impact of these unique amino acid substitutions was predicted using a psi-BLAST based computational tool: SIFT (Sorting Intolerant From Tolerant) (Kumar et al., 2009).

### Data Availability

Sequence data for *P. cristatus* has been deposited in Short Read Archive under project number SRP083005 (BioProject accession: PRJNA040135, Biosample accession: SAMN05660020) and accession codes: SRR4068853 and SRR4068854. We have provided the draft genome assembly, assembly statistics, gene-set and their annotations, alignments, and individual gene tress at our web server^1^. This data is publicly available for download with proper citation as mentioned on the web page.

## Results

### Genomic Features of Indian Peacock

The whole genome sequencing of peacock yielded 153.7 Gb of sequence data (∼136× genomic coverage; **Supplementary Data S1** and **Supplementary Figures S2, S3**). High-quality sequence reads were used to generate a draft genome assembly of an estimated genome size of 1.13 Gb using ABySS, Gapcloser, and Agouti (**Supplementary Data S2**). The *de novo* genome scaffold and contig N50s were 25.6 and 19.3 Kb, respectively (**Supplementary Data S2**). BUSCO scores assessed the genome assembly to be 77.6% complete (S: 63.4%, D: 14.2%) and predicted 13.5% as partial, and 8.9% as missing BUSCOs (**Supplementary Data S3**). The comparison of the BUSCO scores and assembly statistics with the other bird genomes is provided in the **Supplementary Datas S4, S5**. Using *ab initio*-based approach, 25,963 candidate coding sequences were identified in peacock. These genes were validated using the homology-based approach, and a final set of 24,831 transcripts and 15,970 protein-coding genes was constructed for peacock. Out of these protein-coding gene sequences of peacock, the pair-wise orthologs for 18,314, 10,232, 9,900, 9,097, and 9,887 genes could be identified in chicken, turkey, flycatcher, zebra finch and duck genomes, respectively. A total of 5,907 combined orthologs could be identified across all the six bird species.

**FIGURE 1:**
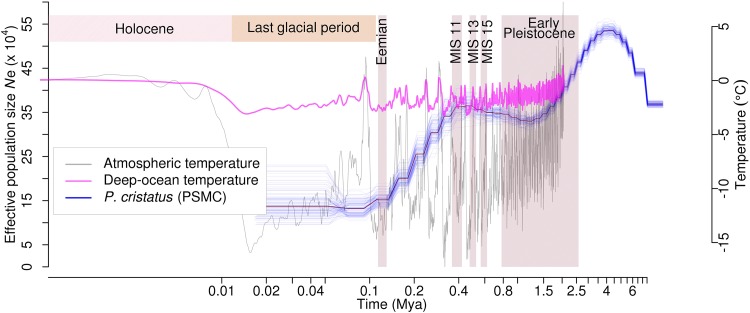
Effective population size (N_e_) estimated from PSMC analysis for Peacock. The changes in effective population size (N_e_) for the peacock is shown as the blue line plot. The thick line represents the consensus, and the thin light line corresponds to 100 bootstrapping rounds. Atmospheric and deep ocean temperatures from Bintanja and Van De Wal (2008) have been overlaid.

A total of 213 tRNAs, 236 snoRNAs, and 540 miRNAs were also identified (**Supplementary Data S6**). Since many miRNAs also bind to the target 3′-UTR with imperfect complementarities and function as translational repressors, miRNA targeting sites in the upstream regions of the genes were identified for peacock and other five bird genomes. A total of 306 miRNAs targeting 2,379 genes were identified using this approach in peacock. These genes predominantly belonged to the functional categories such as signal transduction, protein modifications, and transcription (**Supplementary Figure S4**) for all the birds including peacock. The peacock genome was found to have less repetitive DNA (8.62%) as compared to chicken (9.45%) (**Supplementary Data S7**). The comparison of single nucleotide variants (SNVs) between chicken and peacock revealed 2,051,161 heterozygous SNVs at a rate of 2.05 SNV per Kb. The observed SNV rate in peacock was closer to turkey in comparison to the other avian species (**Supplementary Text S4** and **Supplementary Data S8**). PSMC analysis suggested that the peacock suffered at least two bottlenecks (around four million and 450,000 years ago), which resulted in a severe reduction in its effective population size (**Figure 1**). It was also interesting to note that the results of PSMC analysis of peacock were similar to the demographic history of the tropical bowerbird and turkey vulture that show long-term decrease in the effective population size (Nadachowska-Brzyska et al., 2015), perhaps because all three birds are native to the tropical rain forests.

### Comparative Genomics Analysis

Although more than 50 bird genomes have been sequenced so far, yet comprehensive and curated gene-set is available only for a limited number of bird genomes at Ensembl (Zerbino et al., 2017). Thus, the comparative genomics analysis was performed using only the high-quality genome assemblies of species relatively closer to pheasants, which were available at Ensembl. In this study, a total of six species were included: peacock, chicken, turkey, duck, zebra finch, and flycatcher.

#### Resolving the Phylogenetic Position of Peacock

The phylogenetic position of peacock was determined using a maximum likelihood-based phylogenetic analysis performed using the coding sequences of 5,907 orthologous genes identified from the six bird genomes: peacock, chicken, turkey, duck, flycatcher and zebra finch genomes. The maximum likelihood phylogeny was constructed from the concatenated alignments of orthologous genes with a bootstrap value of 1,000.

**FIGURE 2:**
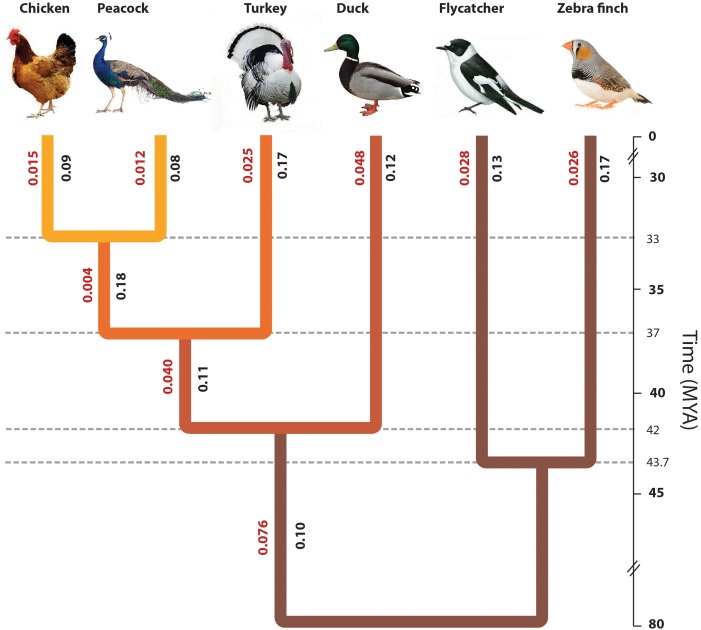
Phylogenetic position of peacock with respect to other bird genomes. The phylogenetic tree constructed from the concatenated alignments of the orthologous genes across all six species. The divergence time of different bird species was determined using the TimeTree database (Hedges et al., 2006), which is based on the published reports of molecular and fossil data. The origin of turkey was estimated to be ∼37.2 MYA, whereas the origin of peacock and chicken was estimated to be ∼32.9 MYA. The original phylogeny from the data had a polytomy due to which the split point between zebra finch and flycatcher could not be identified. However, for the sake of correct visual interpretation the divergence point between flycatcher and zebra finch (∼44 MYA) was identified using the TimeTree database. The values mentioned in Red are the branch length values and the values mentioned in Black are the phylogenetically corrected branch-specific ω or dN/dS values.

The phylogeny for these taxa is well-studied, and *Passeriformes* and *Galliformes* orders are known to form the valid groups since 19th century. Later on, *Galliformes* and *Anseriformes* were found to be monophyletic based on molecular data and formed a new group *Galloanserae* (Sibley et al., 1988; Mayr, 2011). From the phylogenetic tree obtained in this study, it is apparent that Galliformes and Anseriforms are monophyletic, forming the known group *Galloanserae* (**Figure 2**). Moreover, peacock was found closer to chicken than turkey in the *Galliformes* order (**Figure 2**). These results are in agreement with a number of previous studies such as Wang et al. (2013). However, these results contradict the studies based on the retroposon insertion phylogeny and the chromosomal evolution study (Stock and Bunch, 1982; Shibusawa et al., 2004; Kaiser et al., 2007). Therefore, one could argue that the position of peacock with respect to chicken and turkey remains somewhat ambiguous. In total, there are three tree topologies possible with respect to the three taxa:

(t1) ((Ficedula,Taeniopygia), Anas, (Meleagris, (Gallus, Pavo)));
(t2) ((Ficedula,Taeniopygia), Anas, (Gallus, (Meleagris, Pavo)));
(t3) ((Ficedula,Taeniopygia), Anas, (Pavo, (Gallus, Meleagris))).

We tested among these trees using our concatenated alignments of orthologs, finding (t1) with a bootstrap percent value of 100%. Thus, our result was congruent with Wang et al. (2013), who found t1 in their analysis of six nuclear intron regions and two mitochondrial regions. This result differs from the other studies, since it did not agree with the t2 topology observed in the retroposon insertion and the chromosomal evolution based studies (Shibusawa et al., 2004; Kaiser et al., 2007), or the t3 topology observed by neighbor-joining tree analysis of a few nuclear intron regions and mitochondrial regions (Wang et al., 2013).

We constructed the maximum likelihood phylogeny for each of the 5,907 orthologs and estimated the Robinson and Foulds distance (Robinson and Foulds, 1981) of these individual gene trees with the above mentioned three tree topologies. A total of 2,581 (43.7%) orthologs showed the support for topology t1, 1,674 (28.3%) orthologs showed the support for topology t2, 1,376 (23.3%) showed the support for topology t3, and 263 (4.4%) showed support for topology very different from t1, t2, and t3. The histogram for the RF distance from the topology t1, t2, and t3 is shown in **Supplementary Figure S5**. However, the categorization of these genes supporting different topologies based on their molecular function GO categories did not show any pattern of enrichment (**Supplementary Figure S6**). We observed that the maximum support is obtained for the t1 topology. A similar topology was reported by different studies using different kinds of data such nuclear regions, intronic regions, mitochondrial regions, and genome-wide sampled ultraconserved elements (Kimball and Braun, 2014; Sun et al., 2014; Hosner et al., 2015, 2017; Wang et al., 2017a). Although a recent report have shown that the data-type used for constructing the phylogeny may have a major impact on the tree estimate, and have observed that the phylogeny based on coding regions and non-coding regions were very different (Reddy et al., 2017), the most supported topology “t1” in this study is also supported by the phylogenies constructed using different types of data such as non-coding intron (Kimball and Braun, 2014), ultraconserved elements (Sun et al., 2014; Hosner et al., 2015, 2017; Wang et al., 2017a), and 3′-untranslated regions (Bonilla et al., 2010). This suggests the robustness of the t1 topology-based phylogeny.

The phylogenetic tree reported in **Figure 2** had a polytomy due to which the divergence point between the two Passerines, flycatcher and zebra finch, could not be estimated. Thus, the divergence point between flycatcher and zebra finch was derived from the species divergence time from TimeTree (Hedges et al., 2006). The phylogeny of the other birds used in this study is already well-resolved, and thus, the aim of this study was to resolve the phylogenetic position of Peacock in the *Gallliformes* order with respect to the other known genomes. The divergence time is derived from the TimeTree database (Hedges et al., 2006), where the divergence time of peacock and chicken is ∼33 MYA, the divergence of turkey and common ancestor of peacock and chicken is ∼37 MYA, divergence between duck and common ancestor of Galliformes bird species is ∼78 MYA, and the divergence between zebra finch and flycatcher is ∼44 MYA (Hedges et al., 2006). The early divergence of Galliformes and Anseriformes is supported by the studies where the origin of Galliformes and Anseriformes is reported to be during the period of Paleocene (Claramunt and Cracraft, 2015; Cracraft et al., 2015). This early divergence of Galliformes and Anseriformes is also supported by the Next-generation DNA sequencing data from 259 loci (Prum et al., 2015). The divergence time of peacock and chicken, and the divergence of the common ancestor of peacock and chicken with the turkey, was suggested to be during the period of Oligocene (∼23–35 MYA) based on fossil and DNA sequence (nuclear and mitochondrial) data (Wang et al., 2017b), which supports the divergence time from TimeTree. However, in a recent study based on four mitochondrial and six nuclear genes, the divergence of peacock and chicken was estimated to be ∼25–30 MYA, which is slightly earlier than the TimeTree reported divergence time of ∼33 MYA (Cai et al., 2018).

The mitochondrial genome, which evolves independent of the nuclear genome, was also used to infer the phylogenetic relationships using the complete mitochondrial genome sequences of peacock and 22 species from five different classes of Chordates, which included Aves, Mammalia, Reptilia, Actinopterygii, and Amphibia (**Supplementary Figure S7A**). We observed the same anomaly (finding the earliest divergence between the *Passeriformes* and all of the other birds) in our mitochondrial tree of 22 species that has been attributed to difficulties in analyzing the mitochondrial genomes (Braun and Kimball, 2002). In fact, this topology has also been observed previously with the mitochondrial genomes since late 1990’s (Mindell et al., 1999). However, the RY-coding (A and G coded as R, C and T are coded as Y) has been shown to reduce the problem to some extent (Braun and Kimball, 2002; Slack et al., 2007). Thus, we have also constructed the mitochondrial phylogeny for these 22 species with RY-coding and Binary-coding (A and G are coded as 0, C and T are coded 1). The RY and Binary coded phylogenies are mentioned in the **Supplementary Figures S7B,C**. Both, the RY-coded and Binary-coded phylogenies, showed the earliest divergence between a clade that comprises Galloanseae as well as the only Palaeognathae included in our study and Neoaves (the remaining living birds). This topology is more consistent with the well-accepted modern bird phylogeny (Jarvis et al., 2014) that places the root of living birds between Palaeognathae and Neognathae (*Galloanserae* and Neoaves). Thus, in this study also, the RY or binary coding appear to better resolve the mitochondrial phylogeny as reported in earlier studies (Braun and Kimball, 2002; Slack et al., 2007).

Further, the mitochondrial phylogeny was also constructed separately for the six bird species (**Supplementary Figure S8**). The phylogenetic positions of the six bird species were found similar in the phylogenetic tree obtained from the concatenated alignments of orthologs and the phylogenetic tree obtained from the mitochondrial genome of these six birds (**Figure 2** and **Supplementary Figure S8**). The mitochondrial phylogeny was consistent with the most supported nuclear topology t1, genome-wide ultraconserved elements based phylogeny (Sun et al., 2014; Hosner et al., 2015, 2017; Wang et al., 2017a), and with many previous reports of mitochondrial phylogeny for Galliformes order (Kan et al., 2010; Shen et al., 2010; Meiklejohn et al., 2014).

Overall, the phylogenetic analysis carried out using nuclear-genes and mitochondrial genomes in this study revealed that peacock is closer to chicken as compared to turkey (supports the topology t1). This resolves the phylogenetic position of peacock with respect to the well-studied modern bird phylogeny (Jarvis et al., 2014).

#### Assessing the Rate of Divergence in Functional Genome

Other than the branch length values derived from the maximum likelihood phylogeny, the ω or dN/dS values (phylogenetically corrected and pair-wise) will be crucial in understanding the evolutionary rate of divergence. The phylogenetically corrected analysis is usually more preferred but could include only 5,907 orthologs, however, the pair-wise analysis allowed the inclusion of more than 9,000 orthologs for each species pair. Therefore, the phylogenetically corrected branch-specific ω or dN/dS values were derived for the concatenated alignments for each of the extant taxa and for the ancestral nodes in the phylogeny. The values were lower for chicken and peacock in comparison to the other extant bird genomes (**Figure 2**). Furthermore, the pair-wise ω or dN/dS values were estimated for the individual orthologous alignments. The genes were categorized into different KEGG categories, and the median value of ω or dN/dS was determined for each KEGG category (**Supplementary Text S5**). The distribution of the median values plotted for the different pairs showed that the categories involved in signal transduction and transcription regulation have evolved at a very different rate in peacock in comparison to other birds (**Supplementary Figures S9–S11**).

#### Gene Gain/Loss

The analysis of gene gain/loss in gene families was also performed for the six bird genomes namely peacock, chicken, turkey, duck, flycatcher, and zebra finch. To identify the gene families and assign different genes to these families, orthologous gene clustering was performed. This allowed for the identification of different gene families, single-copy orthologs, multi-copy orthologs, and species-specific orthologs for each bird species (**Supplementary Text S6**, **Supplementary Data S9**, and **Supplementary Figure S12**). For phylogenetic correction, the phylogenetic tree topology t1 was utilized, which is having maximum support in terms of bootstrap values and tree distance from individual gene trees.

**FIGURE 3:**
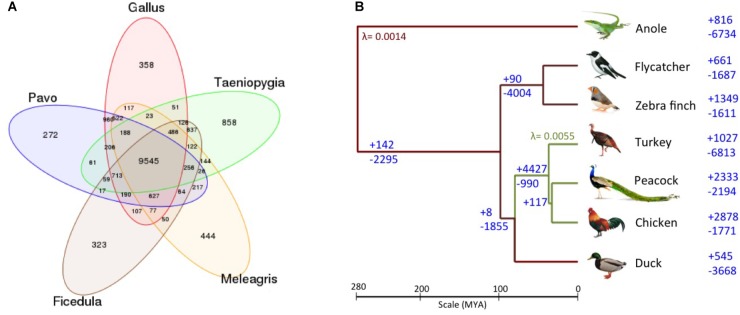
**(A)** Venn diagram of gene families identified using TreeFam. A total of 9,545 gene families were common among the five bird genomes. 522 gene families were unique to the genus (*Pavo, Gallus*, and *Meleagris*) of *Galliformes* order, whereas, 637 gene families were unique to the genus (*Ficedula* and *Taeniopygia*) of *Passeriformes* order. **(B)** Gene gain/loss in the six avian species and anole. The number of gene gain (+) and loss (-) are mentioned on the right of the taxa (branches), for the six avian species and an outlier green anole. The gene gain and loss were calculated using CAFE two-lambda model with λ = 0.0055 for *Galliformes* and λ = 0.0014 for the rest of the tree.

The Venn diagram of the gene families for these bird genomes is shown in **Figure 3A**. Additionally, the phylogenetic tree with the gene gain and gene loss values for the six bird genomes and the outlier green anole is displayed in **Figure 3B**. It is apparent that the common ancestor to the birds in the phylogenetic tree show a loss of 2,295 genes, which is also supported by a previous report mentioning the loss of around 2,000 genes in the ancestor as compared to other vertebrate lineages (Huang et al., 2013; Lovell et al., 2014). However, such observations needs further confirmation as they could arise as an artifact of poor genome coverage in the GC-rich regions and incomplete genome assemblies (Bornelöv et al., 2017), leading to an over or under-estimation of gene counts due to fragmentation of genes on multiple contigs and gaps in the assembly (Denton et al., 2014). We observed that contraction has been more prominent in comparison to expansion for the common ancestor of Galliformes and Anseriformes, and the same pattern has also been observed for turkey and duck (**Figure 3B**). These observations corroborates with the previous study (Huang et al., 2013). However, an opposite pattern of expansion in gene families was observed for peacock and chicken (**Figure 3B**). The top 20 protein families featuring gain and loss in the peacock genome are listed in **Supplementary Datas S10, S11**. Genes from different bird species belonging to these selected families are mentioned in **Supplementary Table S1**. All the top 20 families that showed contraction in peacock, belonged to the hypothetical protein family or had no known function. Whereas, among the top 20 categories that showed expansion in peacock, only one family had a known function (i.e., immune system development and regulation).

#### Identification of Molecular Pathways With Adaptive Divergence

The genome-wide adaptive divergence and evolution of peacock genes were evaluated by three different methods: (i) pair-wise dN/dS estimation: to understand the rate of divergence of functional genes in different species pairs, (ii) positive selection analysis: to identify the genes and corresponding functional categories/pathways evolving under positive selection in peacock, and (iii) unique substitution analysis: to identify genes with unique amino acid changes in peacock. Furthermore, the functional impact of these unique amino acid substitutions was predicted using SIFT (Sorts Intolerant From Tolerant) prediction tool (Kumar et al., 2009). The details of the above analyses and the KEGG and eggNOG annotation of resultant genes are provided in the **Supplementary Text S7**.

The pair-wise dN/dS values were determined for all the pair-wise orthologs of peacock. A total of 74 genes showed the values >1, of which 25 genes had values above five indicating possible false positives, and 22 genes had values between 1.0 and 1.2, which may indicate the relaxation of constraint rather than positive selection, and thus, these 47 genes were not considered for the functional interpretation (**Supplementary Table S2**). The remaining genes with higher pair-wise dN/dS values are considered to be fast evolving in peacock with respect to the other species. The combined orthologs were used for testing positive selection in peacock.

A total of 437 genes displayed signs of positive selection identified using branch-site model-A, and the statistical significance was evaluated using likelihood ratio tests with FDR *q*-value threshold of 0.1 (10% false discovery rate in multiple testing correction) (**Supplementary Table S3**). A total of 417 genes contained amino acid sites, which were under significant positive selection based on the Bayesian Empirical Bayes values. Unique amino-acid substitutions in peacock were found for 3,238 genes, of which the substitutions in 116 genes were predicted to affect the protein function based on SIFT (Sorting Intolerant from Tolerant) analysis (**Supplementary Tables S4, S5**). In total, 99 genes showed positive selection and unique amino acid substitutions that may affect the protein function predicted using SIFT and are referred to as the genes with “multiple signs of adaptation” (MSA) in this study (**Supplementary Table S6**).

The functional analysis revealed the role of these genes in key cellular processes such as cell proliferation and differentiation (MAPK, RAS, PI3K-Akt, ErbB, Hippo, Rap1, and Jak-STAT signaling, Wnt signaling, calcium signaling and adrenergic signaling in cardiomyocytes) and immune response (T cell receptor, Toll-like receptor signaling, NOD-like receptor signaling, complement and coagulation cascade and chemokine-chemokine signaling). In addition, multiple genes involved in early development pathways such as TGF-β, Wnt/β-catenin, FGF, and BMP signaling also showed adaptive sequence divergence in peacock. These cellular processes and pathways regulate key features such as early development, feather development, bone morphogenesis, skeletal muscle development, metabolism, and immune response (**Supplementary Table S7**).

An interesting observation was made from signaling pathways such as Wnt, Rap1, Ras, Jak-Stat, and cAMP-mediated GPCR signaling. It was observed that the ligand and/or receptor, and in some cases the final effector genes showed adaptive evolution, whereas the genes involved in the intermediate signal transduction processes remained conserved perhaps due to their common role in multiple signaling pathways (**Supplementary Text S8**). Another interesting observation was that in several interacting protein pairs, both the interacting proteins showed sequence divergence in the peacock (**Supplementary Text S9**) hinting toward their similar evolution (Moyle et al., 1994). These protein pairs were majorly involved in early development pathways such as Wnt, BMP, and TGF-β signaling, cell cycle regulation, DNA replication, GPCR signaling, and gene expression regulation (**Supplementary Table S8**).

### Assessing the Evolution of Genes Involved in Specific Phenotypes

#### Adaptive Evolution of Early Developmental Pathways

The early developmental pathways, which are crucial in guiding the embryonic development in birds such as TGF-β, Wnt, FGF, and BMP signaling, showed adaptive divergence in peacock (Klaus and Birchmeier, 2008). Among these pathways, the TGF-β pathway is known to regulate the cartilage connective tissue development (Loveridge et al., 1993), and also functions as an activator of feather development in birds. In this pathway, TGFBR3 gene showed MSA, and TGF-β3 preproprotein, TGFBRAP1, and TAB3 genes showed multiple unique substitutions. The Wnt signaling pathway is involved in development, regeneration, aging process (Brack et al., 2007; Klaus and Birchmeier, 2008), and also regulates the initial placement of feather buds and their consolidation within the feather field (Lim and Nusse, 2013). Multiple regulators of Wnt signaling such as WNT2, WIF1, and DKK2 genes had positively selected amino acid sites and showed signs of adaptive evolution. The WIF1 and DKK2 genes also harbored multiple unique substitutions. Furthermore, the DKK2 and WNT2 genes were found to be positively selected in peacock. APCDD1 gene, which is an inhibitor of Wnt signaling pathway, showed MSA. The Bone Morphogenetic Protein (BMP) signaling is involved in the development of skeletal muscles, bone and cartilage connective tissue (Nie et al., 2006; Nishimura et al., 2012), neurogenesis (Groppe et al., 2002), and feather formation and patterning. Multiple genes such as BRK-3, BMP5, BMP3, BMP10, and CRIM1, which are involved in the regulation of BMP pathways and the corresponding early development, showed unique substitutions that may affect their function in cellular pathways as compared to the other birds.

In addition, the Notch-2 receptor gene of Notch-Delta signaling, which is involved in growth and patterning of feather buds, early development of sensory organs (Crowe et al., 1998), and terminal muscle differentiation, also showed five unique substitutions. Unique substitutions were also found in the FGFR3 receptor gene and FGF23 genes, which are part of the FGF signaling involved in limb and skeletal muscle development, feather development and morphogenesis, and regulation of feather density and patterning (Pownall and Isaacs, 2010).

Taken together, the multiple signs of evolution observed in the genes of early development pathways in peacock suggest the adaptive divergence in the early development processes, including feather, bone and skeletomuscle development.

#### Peacock Feathers: Clues From Early Development Genes

Among the distinctive features of a peacock, the large and decorative feathers attract the most attention; particularly the long train, which is useful for their courtship behavior. The feather development in birds is primarily guided by the continuous reciprocal interactions between the epithelium and mesenchyme (Chuong et al., 2000). The analysis of the curated set of 2,146 feather-related genes (**Supplementary Text S10**) involved in feather development revealed that the activators of feather development including FGF, Wnt/β-catenin and TGF-β and, the inhibitors such as BMP and Notch-delta showed sequence divergence in peacock in comparison to the other bird genomes. The observed divergence in genes related to feather development provides useful genomic clues for the peculiar patterning and structure of peacock feathers.

#### Adaptive Evolution in Immune-Related Genes

In birds, the rate of sequence divergence in immune-related genes is usually higher than the other genes primarily due to the co-evolution of host–pathogen interactions (Ekblom et al., 2010). Several genes involved in the development of immune system and modulation of immune response have shown sequence divergence and signs of adaptive evolution in the peacock genome.

Multiple components of the innate immune system such as complement system and pathogen recognition system showed adaptive evolution. The C5 protein involved in the recruitment of cellular component of the immune system at the site of infection showed five unique substitutions. The α-subunit of C8 protein involved in forming the membrane attack complex (MAC) (Serna et al., 2016) showed MSA. Additionally, the CSF-1R gene, which is crucial for macrophage survival, differentiation, and proliferation (Pixley and Stanley, 2004), showed positive selection with positively selected sites and unique substitutions. Different components of NF-κB signaling such as MYD88, TRADD, SIGIRR, MAP3K14, and TLR5, which regulate the immune response against infections (Kaisho and Akira, 2006), showed signs of adaptations. The MYD88 protein, which is a part of Toll-like receptors (TLRs) mediated signaling, showed MSA and higher divergence from chicken in comparison to turkey among the species of the Galliformes order. Similarly, the genes TRADD, SIGIRR, MAP3K14, and TLR5 showed multiple unique substitutions. Furthermore, the pattern recognition receptors such as NLRC3, which regulates innate immune response by interacting with stimulators of interferon genes (Zhang L. et al., 2014), showed positive selection with positively selected sites and unique substitution.

Several genes regulating the T and B-cell response of the adaptive immune system also displayed adaptive evolution in peacock. The SPI-1 gene involved in B and T cell development by regulating the expression as well as alternative splicing of target genes (Hallier et al., 1998) showed MSA. Further, the different T-cell receptors and signaling proteins involved in T-cell activation such as SDC4, FLT4, NFATC3, and IL12B subunit showed sequence divergence and MSA in peacock. CTLA4 gene, which is a negative regulator of T-cell response (Walunas et al., 1994), also showed multiple unique substitutions. In addition, the gene family SSC4D involved in the development of immune system and the regulation of both innate and adaptive immunity (Asratian and Vasil’eva, 1976) showed expansion in peacock in comparison to chicken (**Supplementary Data Sheet S1**). Other than the genes discussed above several other genes having implications for immune system development and response have shown adaptive evolution in peacock and are discussed in the **Supplementary Text S11**.

Taken together, it appears that the adaptive evolution of immune-related genes in peacock has occurred primarily in the components of innate immunity such as complement system, pattern recognition receptors, and monocyte development, and in the components of adaptive immunity such as T-cell response. It suggests that the immune system-related genes in peacock genome have adaptively evolved, which has also been observed in genome-wide scans of other species (Kosiol et al., 2008; Fumagalli et al., 2010; Deschamps et al., 2016; Van Der Lee et al., 2017).

Few more genes with known phenotypic impact and which showed MSA in peacock are discussed in **Supplementary Texts S12, S13**.

## Discussion

Indian Peacock (*P. cristatus*) has been a model species in the emergence of the sexual selection theory and its distinctive phenotypes have intrigued biologists for decades. The unique sexual characteristics and ornamental features of peacock provide impetus for deciphering the genomic changes underlying such phenotypes. Thus, revealing the genome sequence of this unique species with a comprehensive comparative genomic analysis provides a useful reference and research leads to the ornithologists and evolutionary biologists.

The achieved BUSCO scores and contig N50 values of the peacock genome were on par or better than many other bird genome assemblies including some of the recently published genomes from *Galliformes* order (Rhinoceros hornbill, Yellow-thoated sandgrouse, Kea, Chinese bamboo partridge, Mikado Pheasant etc.) (Zhang G. et al., 2014; Lee et al., 2018; Tiley et al., 2018; Wu et al., 2018). For peacock, two genomic libraries with similar insert sizes were used along with a high (136X) sequence coverage. It is known that the usage of multiple insert size libraries (preferably with broad insert sizes) is needed to achieve higher N50 values but it significantly increases the sequencing cost. In the case of peacock, using two similar insert sizes and a higher coverage, reasonably good N50 and BUSCO scores were achieved with low-sequencing cost making it amenable for comparative analysis.

**FIGURE 4:**
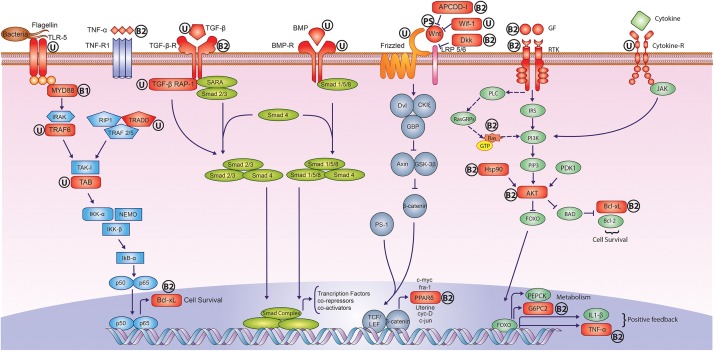
Adaptively evolved signaling pathways in peacock genome. The genes highlighted in Red color showed signs of adaptive evolution such as positive selection and unique substitution. The symbol “U” stands for only unique substitution(s), the symbol “PS” stands for only positive selection, the symbol “B1” stands for unique substitution(s) and also harbors the positively selected amino acid residues but overall genes do not show any positive selection, and the symbol “B2” stands for unique substitution(s) and positive selection. It is apparent that the receptors, ligands and regulators of early development pathways such as Wnt, TGF-β, and BMP, showed adaptive sequence divergence in peacock. In the case of NF-κB, cytokine and growth factor signaling pathways, the proteins involved in intermediate signal transduction also showed adaptive sequence divergence. Individual pathways are color coded separately.

The phylogenetic analysis of peacock genome using the orthologous gene sequences revealed that it is closer to chicken in comparison to turkey. However, the phylogenetic position of peacock using the complete genome-wide data including non-coding parts such as introns is yet to be resolved. The most significant results emerged from the adaptive sequence divergence analysis, where a major fraction of genes involved in early development and immune system showed multiple signs of adaptive evolution (**Figure 4**). Similarly, the genes involved in the early development of feathers showed signs of adaptive evolution in the feather-specific gene set. In addition, the adaptive divergence observed in the genes involved in bone morphogenesis and skeletal muscle development perhaps explain the large body dimensions, stronger legs and spurs, and the ability to take short flights despite of a long train. Taken together, the evolution in the early development genes emerges as a prominent factor for explaining the molecular basis of the phenotypic evolution for Indian peacock.

Differential species-specific immunity against pathogens has been observed earlier in many species. In the case of peacock, the resistance to a new viral strain Massachusetts prototype like coronavirus, which is pathogenic to chicken and turkey, also appears to be a case of species-specific immunity (Sun et al., 2007). This could perhaps be attributed to the adaptive divergence observed in the components of the innate immune system (complement and pathogen recognition system), adaptive immune response (B and T cell development), and other genes responsible for the overall immune system development. Moreover, the adaptive evolution observed for immune genes in peacock also appears consistent with the predictions of Hamilton–Zuk hypothesis (Hamilton and Zuk, 1982; Balenger and Zuk, 2014). Though the results were obtained from the comparative genomic analysis of peacock, some of the insights are applicable to the other related species in the pheasant group.

Although different filters have been used to reduce the false positives and false negatives, for instance filtering the genes with >10% of ambiguous bases, excluding the genes with very high (>5) or nearly neutral (1.0–1.2) dN/dS values, and by applying stringent statistical tests, yet some analyses such as positive selection are prone to false positives. Therefore, the results should be interpreted with care and the identified positively selected genes should be considered as the candidate genes. This problem is common in genome-wide analysis and some approaches to address the problem can inflate the false positives, whereas others may increase the rate of false negatives (Redelings, 2014; Tiley et al., 2018); that said, the inferences provided by such methodology are worthwhile and have clearly provided insights into peacock evolution.

The comparative genomic analysis presented in this work provides novel insights on the phenotypic evolution of Indian Peacock and the genomic clues from this study will serve as leads for further studies to decipher the genotype–phenotype interactions for peacock. In addition, this study will also help in devising better strategies for the management and conservation of peacock population, which is susceptible to decline mainly because of habitat deterioration, poaching for train-feathers, use of pesticides and chemical fertilizers.

## Author Contributions

VKS conceived and coordinated the project. RS prepared the DNA samples, performed sequencing, and the molecular sexing assay. AG performed the *de novo* and reference-based genome assembly. PM, AKS, AG, and SKJ performed the genome annotations. SKJ and PM performed the phylogenetic tree analyses. SKJ performed the dN/dS, positive selection, and statistical analysis. SKJ, AG, and AR performed the unique substitution and SIFT analyses. PM performed the gene gain/loss analysis. SKJ, VP, and AG created figures. SKJ, AG, VKS, NV, and AS analyzed the data and wrote the manuscript. All the authors have read and approved the final manuscript.

## Conflict of Interest Statement

The authors declare that the research was conducted in the absence of any commercial or financial relationships that could be construed as a potential conflict of interest.

## Footnotes

1 http://metagenomics.iiserb.ac.in/peacock/data.php
