# Supplementary material for: Genome Sequence of Peacock Reveals the Peculiar Case of a Glittering Bird
Source: Front Genet. 2018 Sep 19;9:392. doi: 10.3389/fgene.2018.00392 (PMC6156156; doi:10.3389/fgene.2018.00392)
Supplement: Supplementary file 1 [file Data_Sheet_1.docx]

**SUPPLEMENTARY INFORMATION**

**Title:** Genome Sequence of Peacock Reveals the Peculiar Case of a Glittering Bird

**Authors:** Shubham K. Jaiswal^+1^, Ankit Gupta^+1^, Rituja Saxena^1^, Vishnu Prasoodanan P. K.^1^, Ashok K. Sharma^1^, Parul Mittal^1^, Ankita Roy^1^, Aaron B.A. Shafer^3^, Nagarjun Vijay^2^, Vineet K. Sharma*^1^

**Affiliation:**

^1^Metagenomics and Systems Biology Group, Department of Biological Sciences, Indian Institute of Science Education and Research Bhopal, India

^2^Computational Evolutionary Genomics Lab, Department of Biological Sciences, Indian Institute of Science Education and Research Bhopal, India

^3^Forensic Science and Environmental & Life Sciences, Trent University, Canada

*Corresponding Author email:

Vineet K. Sharma - vineetks@iiserb.ac.in

†These authors contributed equally to this work

Email addresses of authors:

Shubham K. Jaiswal – [shubhamj@iiserb.ac.in](mailto:shubhamj@iiserb.ac.in), Ankit Gupta - [ankitg@iiserb.ac.in](mailto:ankitg@iiserb.ac.in), Aaron B.A. Shafer - [aaronshafer@trentu.ca](mailto:aaronshafer@trentu.ca), Rituja Saxena – [ritus@iiserb.ac.in](mailto:ritus@iiserb.ac.in), Vishnu Prasoodanan P. K.- [vishnup16@iiserb.ac.in](mailto:vishnup16@iiserb.ac.in)**,** Ashok K. Sharma – [ashok@iiserb.ac.in](mailto:ashok@iiserb.ac.in)**,** Parul Mittal - [parulm@iiserb.ac.in](mailto:parulm@iiserb.ac.in), Ankita Roy – [rankitauga@gmail.com](mailto:rankitauga@gmail.com), Nagarjun Vijay - [nagarjun@iiserb.ac.in](mailto:nagarjun@iiserb.ac.in), Vineet K. Sharma - vineetks@iiserb.ac.in

**SUPPLEMENTARY DATASHEETS**

**Supplementary Data S1: Summary of the sequence data of peacock genome**

| Paired-end Insert Size | Read Length | Number of Reads | Total Data | Sequence Coverage |
| --- | --- | --- | --- | --- |
| 550 bp and 300-1200 bp (average 650) | 150 bp | 1,067,644,456 | 153.7 Gb | 136x |

^#^ Sequencing coverage was calculated by assuming the peacock genome size of 1.13 Gb.

**Supplementary Data S2: Summary of *de novo* genome assembly of peacock.**

| Category | Scaffolds | Contigs |
| --- | --- | --- |
| Total number | 98,687 | 123,430 |
| Total size | 1,160,282,751 | 1,137,150,029 |
| Longest size | 286,113 | 154,093 |
| Shortest size | 501 | 148 |
| Mean size | 11,757 | 9,213 |
| Median size | 5,670 | 4,745 |
| N50 length | 25,613 | 19,387 |
| L50 count | 12,614 | 17,044 |
| %A | 28.61 | 29.19 |
| %C | 20.36 | 20.77 |
| %G | 20.39 | 20.81 |
| %T | 28.64 | 29.22 |
| %N | 2.01 | 0.01 |

**Supplementary Data S3: Assembly assessment using BUSCO (Percentile)**

| Total BUSCOs group searched | **4,915** |
| --- | --- |
| Complete BUSCOs | **3,811 (77.6%)** |
| Complete and Single-copy BUSCOs | **3,114 (63.44%)** |
| Complete and duplicated BUSCOs | **697 (14.2%)** |
| Fragmented BUSCOs | **664 (13.5%)** |
| Missing BUSCOs | **440 (8.9%)** |

**Supplementary Data S4: Assembly assessment by comparison of BUSCO (Percentile)**

| Species | Completely Found | Partially Found | Missing |
| --- | --- | --- | --- |
| *P. cristatus* | 3811 (77.6%) | 697 (14.2%) | 440 (8.9%) |
| *S. mikado* | 2495 (82.53%) | 183 (6.06%) | 345 (11.41%) |
| *A. platyrhynchos* | 2444 (80.85%) | 234 (7.74%) | 345 (11.41%) |
| *G. gallus* | 2734 (90.44%) | 106 (3.51%) | 183 (6.05%) |
| *M. gallopavo* | 2438 (80.65%) | 208 (6.88%) | 377 (12.47%) |
| *T. guttata* | 2456 (81.24%) | 229 (7.58%) | 338 (11.18%) |
| *C. japonica* | 2491 (96.33%) | 60 (2.32%) | 35 (1.35%) |



*Values for some species are from the literature ([Lee et al., 2018](#_ENREF_10); [Wu et al., 2018](#_ENREF_19))

**Supplementary Data S5: Assembly assessment by comparison of contig and scaffold N50**

|  |  | Species | Common name | Sequencing depth | Library | Assembly (contig/scaffold N50;total length) |
| --- | --- | --- | --- | --- | --- | --- |
|  |  | ***Pavo cristatus*** | Peacock | 136X | Single | 19.3K/25.6K;1.13 G |
|  |  | ***Bambusicola^1^ thoracicus*** | Chinese Bamboo Partridge | 25X | Single | -/13.2K;1.03 G |
|  |  | ***Antrostomus carolinensis^2^*** | Chuck-will's-widow | 30X | Multiple | 17 K/45 K;1.15G |
|  |  | ***Cariama cristata^2^*** | Red-legged seriema | 24X | Multiple | 17 K/54 K;1.15G |
|  |  | ***Colius striatus^2^*** | Speckled mousebird | 27X | Multiple | 18 K/45 k;1.08G |
|  |  | ***Merops nubicus^2^*** | Carmine bee-eater | 37X | Multiple | 20 K/47 K;1.06G |
|  |  | ***Gavia stellata^2^*** | Red-throated loon | 33X | Multiple | 16 K/45 K;1.15G |
|  |  | ***Balearica regulorum^2^*** | Grey-crowned crane | 33X | Multiple | 18 K/51 K;1.14G |
|  |  | ***Apaloderma vittatum^2^*** | Bar-tailed trogon | 28X | Multiple | 19 K/56 K;1.08G |
|  |  | ***Phalacrocorax carbo^2^*** | Great cormorant | 24X | Multiple | 15 K/48 K;1.15G |
|  |  | ***Phaethon lepturus^2^*** | White-tailed tropicbird | 39X | Multiple | 18 K/47 K;1.16G |
|  |  | ***Phoenicopterus ruber ruber^2^*** | American flamingo | 33X | Multiple | 16 K/37 K;1.14G |
|  |  | ***Podiceps cristatus^2^*** | Great-crested grebe | 30X | Multiple | 13 K/30 K;1.15G |
|  |  | ***Fulmarus glacialis^2^*** | Northern fulmar | 33X | Multiple | 17 K/46 K;1.14G |
|  |  | ***Tyto alba^2^*** | Barn owl | 27X | Multiple | 13 K/51 K;1.14G |
|  |  | ***Tauraco erythrolophus^2^*** | Red-crested turaco | 30X | Multiple | 18 K/55 K;1.17G |
|  |  | ***Cathartes aura^2^*** | Turkey vulture | 25X | Multiple | 12 K/35 K;1.17G |
|  |  | ***Eurypyga helias^2^*** | Sunbittern | 33X | Multiple | 16 K/46 K;1.1G |
|  |  | ***Mesitornis unicolor^2^*** | Brown mesite | 29X | Multiple | 18 K/46 K;1.1G |
|  |  | ***Leptosomus discolor^2^*** | Cuckoo-roller | 32X | Multiple | 19 K/61 K;1.15G |
|  |  | ***Chlamydotis macqueenii^2^*** | MacQueen's Bustard | 27X | Multiple | 18 K/45 K;1.09G |
|  |  | ***Pelecanus crispus^2^*** | Dalmatian pelican | 34X | Multiple | 18 K/43 K;1.17G |
|  |  | ***Pterocles gutturalis^2^*** | Yellow-thoated sandgrouse | 25X | Multiple | 17 K/49 K;1.07G |
|  |  | ***Acanthisitta chloris^2^*** | Rifleman | 29X | Multiple | 18 K/64 K;1.05G |
|  |  | ***Buceros rhinoceros^2^*** | Rhinoceros hornbill | 35X | Multiple | 14 K/51 K;1.08G |
|  |  | ***Nestor notabilis^2^*** | Kea | 32X | Multiple | 16 K/37 K;1.14G |
|  |  | ***Haliaeetus albicilla^2^*** | White-tailed eagle | 26X | Multiple | 20 K/56 K;1.14G |
|  |  | ***Syrmaticus mikado^3^*** | Mikado pheasant | 160X | Multiple | 13.4K/11.4M;1.07 |

**^1^(**[**Tiley et al., 2018**](#_ENREF_18)**); ^2^(**[**Zhang et al., 2014**](#_ENREF_21)**);^3^(**[**Lee et al., 2018**](#_ENREF_10)**)**

**Supplementary Data S6: Summary of ncRNA identified in peacock genome**

| Species | tRNA | miRNA | snoRNA |
| --- | --- | --- | --- |
| Peacock | 213 | 540 | 236 |
| Chicken | 278 | 852 | 229 |
| Duck | 266 | 418 | 217 |
| Turkey | 149 | 226 | 213 |

**Supplementary Data S7: Composition of repetitive sequences of peacock identified using RepeatMasker and compared with the other bird genomes.**

|  | Peacock | | Chicken | | Turkey | | Duck | |
| --- | --- | --- | --- | --- | --- | --- | --- | --- |
| Repeat Type | **Length (Mb)** | **% in genome** | **Length (Mb)** | **% in genome** | **Length (Mb)** | **% in genome** | **Length (Mb)** | **% in genome** |
| DNA | 12.09 | 1.04 | 11.12 | 0.90 | 9.3 | 0.88 | 3.32 | 0.30 |
| LINE | 73.35 | 6.32 | 79.35 | 6.45 | 59.81 | 5.63 | 44.56 | 4.03 |
| LTR | 12.27 | 1.06 | 25.02 | 2.03 | 10.1 | 0.95 | 0.91 | 0.83 |
| SINE | 0.80 | 0.07 | 0.70 | 0.06 | 0.61 | 0.06 | 0.98 | 0.09 |
| Others | 1.14 | 0.09 | 73.05 | 5.94 | 1.04 | 0.10 | 2.21 | 0.21 |
| Unclassified | 0.52 | 0.04 | 0.47 | 0.04 | 0.40 | 0.04 | 0.55 | 0.05 |
| Total | 99.96 | 8.62 | 189.23 | 15.38 | 81.11 | 7.64 | 60.55 | 5.48 |

**Supplementary Data S8: Comparison of the SNV rate in peacock with other avian species.**

| Species | Heterozygous SNV rate per kb* |
| --- | --- |
| Peacock | 2.05 |
| Zebra Finch | 1.4 |
| Turkey | ~1.7 |
| Chicken | 4.5 |

*: The SNV rate for chicken, turkey and zebra finch, are reported from the previous study ([Huxley, 1968](#_ENREF_9)).

**Supplementary Data S9: The results of orthologous gene clustering and gene family assignment for the six avian species and anole.**

| Species | Gene number | Genes in families | Unclustered genes | Family number | Average genes per family |
| --- | --- | --- | --- | --- | --- |
| Ficedula | 15983 | 15190 | 793 | 13399 | 1.13 |
| Taeniopygia | 18204 | 16845 | 1359 | 13501 | 1.25 |
| Meleagris | 16494 | 15956 | 538 | 12908 | 1.24 |
| Gallus | 25697 | 25210 | 487 | 14296 | 1.76 |
| Pavo | 24094 | 23887 | 207 | 13923 | 1.72 |
| Anas | 16353 | 15189 | 1164 | 13421 | 1.13 |
| Anolis | 19176 | 17218 | 1958 | 14675 | 1.17 |

The green anole was used as an out-group in the TreeFam analysis.

**Supplementary Data S10: Top 20 gene families showing contraction in peacock.**

| Gene Family | Flycatcher | Zebra finch | Turkey | Duck | Anolis | Chicken | Peacock | Contraction* |
| --- | --- | --- | --- | --- | --- | --- | --- | --- |
| FAM_20814 | 0 | 1 | 0 | 0 | 0 | 43 | 0 | -43 |
| FAM_20818 | 0 | 1 | 0 | 0 | 0 | 31 | 0 | -31 |
| FAM_6563 | 0 | 0 | 0 | 0 | 0 | 20 | 0 | -20 |
| FAM_20821 | 0 | 0 | 0 | 0 | 0 | 16 | 0 | -16 |
| FAM_14210 | 1 | 1 | 2 | 1 | 1 | 16 | 1 | -15 |
| FAM_20308 | 0 | 0 | 1 | 0 | 0 | 46 | 32 | -14 |
| FAM_118 | 1 | 0 | 1 | 0 | 0 | 13 | 0 | -13 |
| FAM_4189 | 0 | 0 | 2 | 0 | 0 | 13 | 0 | -13 |
| FAM_20864 | 0 | 0 | 0 | 0 | 0 | 12 | 0 | -12 |
| FAM_20310 | 0 | 0 | 0 | 0 | 0 | 23 | 13 | -10 |
| FAM_95 | 0 | 0 | 7 | 0 | 0 | 9 | 0 | -9 |
| FAM_3510 | 0 | 0 | 0 | 2 | 0 | 10 | 2 | -8 |
| FAM_1498 | 35 | 111 | 25 | 24 | 0 | 159 | 153 | -6 |
| FAM_19930 | 0 | 0 | 0 | 0 | 0 | 6 | 0 | -6 |
| FAM_20314 | 0 | 0 | 0 | 0 | 0 | 19 | 13 | -6 |
| FAM_20707 | 0 | 0 | 0 | 0 | 0 | 6 | 0 | -6 |
| FAM_8375 | 0 | 0 | 0 | 0 | 0 | 6 | 0 | -6 |
| FAM_17403 | 0 | 0 | 2 | 0 | 0 | 5 | 0 | -5 |
| FAM_20089 | 0 | 0 | 1 | 0 | 0 | 5 | 0 | -5 |

*the numbers for contraction in peacock are calculated with respect to chicken.

All the gene families observed to be contracted in peacock were hypothetical and had no known functions.

**Supplementary Data S11: Top 20 gene families showing expansion in peacock .**

| Gene Family | Flycatcher | Zebra finch | Turkey | Duck | Anolis | Chicken | Peacock | Expansion* |
| --- | --- | --- | --- | --- | --- | --- | --- | --- |
| FAM_3394 | 0 | 0 | 0 | 1 | 0 | 1 | 6 | 5 |
| FAM_3401 | 0 | 0 | 0 | 0 | 0 | 0 | 5 | 5 |
| FAM_3408 | 0 | 0 | 0 | 0 | 0 | 0 | 5 | 5 |
| FAM_3415 | 0 | 0 | 0 | 0 | 0 | 0 | 5 | 5 |
| FAM_3420 | 0 | 0 | 0 | 0 | 0 | 0 | 5 | 5 |
| FAM_3431 | 0 | 0 | 0 | 0 | 0 | 0 | 5 | 5 |
| FAM_3441 | 0 | 0 | 0 | 0 | 0 | 0 | 5 | 5 |
| FAM_3451 | 0 | 0 | 0 | 0 | 0 | 0 | 5 | 5 |
| FAM_442 | 1 | 2 | 3 | 0 | 1 | 0 | 5 | 5^†^ |
| FAM_4756 | 1 | 1 | 2 | 2 | 1 | 0 | 5 | 5 |
| FAM_13828 | 2 | 17 | 14 | 5 | 0 | 0 | 8 | 8 |
| FAM_3409 | 0 | 0 | 0 | 0 | 0 | 0 | 8 | 8 |
| FAM_3432 | 0 | 0 | 0 | 0 | 0 | 0 | 8 | 8 |
| FAM_3421 | 0 | 0 | 0 | 0 | 1 | 0 | 10 | 10 |
| FAM_3399 | 0 | 0 | 0 | 0 | 0 | 0 | 25 | 25 |
| FAM_3395 | 0 | 0 | 0 | 0 | 0 | 0 | 37 | 37 |
| FAM_3396 | 0 | 0 | 1 | 0 | 0 | 0 | 57 | 57 |
| FAM_3398 | 0 | 0 | 0 | 0 | 0 | 0 | 69 | 69 |
| FAM_3397 | 0 | 0 | 0 | 0 | 0 | 0 | 74 | 74 |

*the numbers for contraction in peacock are calculated with respect to chicken.

^†^ This expanded gene family FAM_442 (Annotation: SSC4D) is involved in the development of immune system, and in the regulation of both innate and adaptive immunity. The other expanded gene families in peacock had no known function.

**SUPPLEMENTARY TABLES**

The supplementary tables are uploaded separately with the manuscript, and their table titles and legends are provided below.

**Supplementary Table S1. The distribution of genes from the six avian genomes in different gene families (IDs)**

**Supplementary Table S2. Genes with higher (>1) pair-wise dN/dS values**

*Reference genome consists of chicken (C), turkey (T), duck (D), flycatcher (F) and zebra finch (Z). The value after the reference genome (separated by underscores) shows the dN/dS value.

**Supplementary Table S3. Genes exhibiting positive selection in peacock**

**Supplementary Table S4. List of genes exhibiting unique substitutions in peacock**

**Supplementary Table S5. List of unique substitutions that may result in change in protein function predicted using SIFT**

***** The column shows the site at which the change in amino acid results in an alteration of protein functionality in peacock with respect to the other avian species. For eg. D778E represents the change in amino acid D which is replaced by E at 778th position in peacock coding gene sequences.

**Supplementary Table S6. Genes exhibiting multiple signs of adaptation (MSA) in peacock**

**^1^** 'Y' represents positive selection for the corresponding peacock gene.

^2^ The alphabetic character mention the amino acid site under positive selection in peacock, followed by the position in peacock gene and corresponding BEB probability values, which are separated by underscores

^3^ The values mentioned show unique substitution of amino acids in peacock as compared to the other avian species. For e.g. D778E represents the change in amino acid D which is replaced by E at 778th position in peacock coding gene sequence.

^4^ The column mentions the unique substitutions that were found to affect the protein functionality with respect to the other avian species. For e.g. D778E represents the change in amino acid D which is replaced by E at 778th position in peacock coding gene sequence.

**Supplementary Table S7. Distribution of genes with unique substitution and signatures of positive selection in eggNOG classes**

The major fraction of genes containing unique substitutions and showing signatures of positive selection belonged to the eggNOG categories: Signal transduction mechanisms (T), Intracellular trafficking, secretion, and vesicular transport (U), Posttranslational modification, protein turnover, chaperones (O) and Transcription (K).

**Supplementary Table S8. Interacting gene-pairs showing similar signs of adaptive evolution in the coding gene sequences.**

PS_US: Positively selected genes, which also harbours unique substitutions. PS: Positively selected genes. PSS: Genes possessing positively selected amino acid sites. DNDS4x: Genes showing dN/dS enrichment by a factor of 4. US: Genes possessing unique substitutions.

**SUPPLEMENTRAY FIGURES**


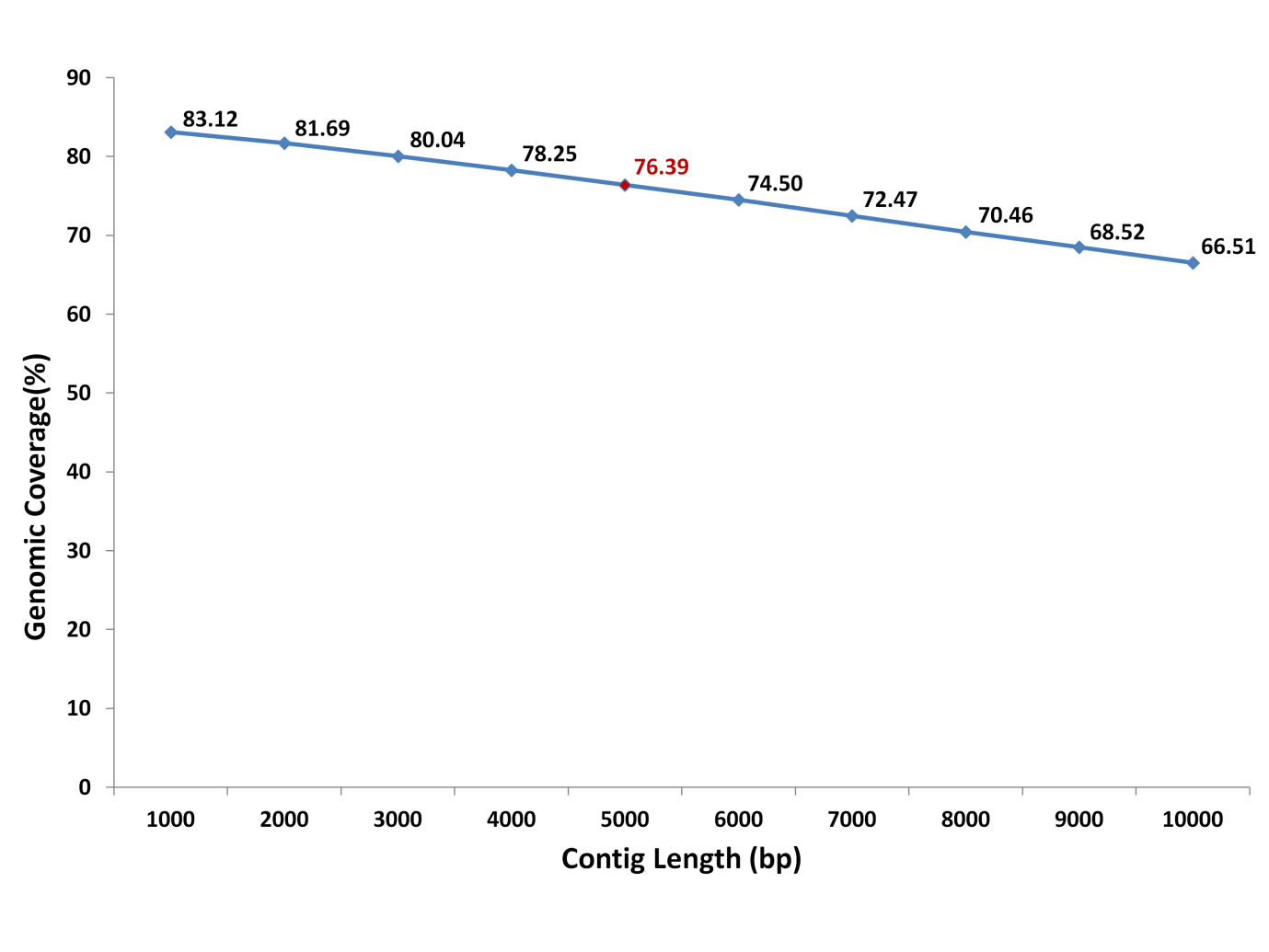


**Supplementary Figure S1.** **Selection of minimum contig length for the PSMC analysis on the basis of percentage of genome covered**

**Supplementary Figure S2:** **Distribution of 21-mer frequency in the corrected PE reads for the estimation of the genome size of peacock.**

The X-axis and Y-axis represents the sequencing depth and the proportion of counts of a K-mer in the total K-mer counts at a given sequencing depth, respectively. Hypothetically, the distribution of bp-mer should follow a Poisson distribution if the sequencing data is free from sequence error, heterozygosity and genome repeats, in general, sequence errors results in a larger proportion of low-depth frequencies. The peak of the 21-mer frequency (M), 53 in this case, in the reads is correlated to the real sequencing depth (N), read length (L), and Kmer length (K), and their relationship can be expressed by the experimental formula, M = N * (L – K + 1) / L. The peacock genome size is estimated 1.01 GB by dividing the total sequence length by the real sequencing depth.

The genome size estimated above did not include the transcriptome data used to achieve the final genome assembly of 1.13 Gb (Supplementary Table 2) using AGOUTI. Additionally, the genome size predicted by flow cytometry of red blood cells of a close relative *Pavo muticus* was 1.31Gb ([Ouyang et al., 2009](#_ENREF_16)). Hence, we used the maximum achieved genome length after AGOUTI assembly (1.13 Gb) as the genome size of peacock for the further analysis.

**Supplementary Figure S3:** **Sequencing depth distributions for peacock genome assembly.**

Peacock whole genome sequence reads were mapped to the assembly by SOAPaligner, with a threshold of two mismatches. The sequencing depth at each position was counted according to the corresponding number of reads in the peacock assembly.

**Supplementary Figure S4:** **The classification of miRNA target genes into eggNOG categories for the six avian species.**


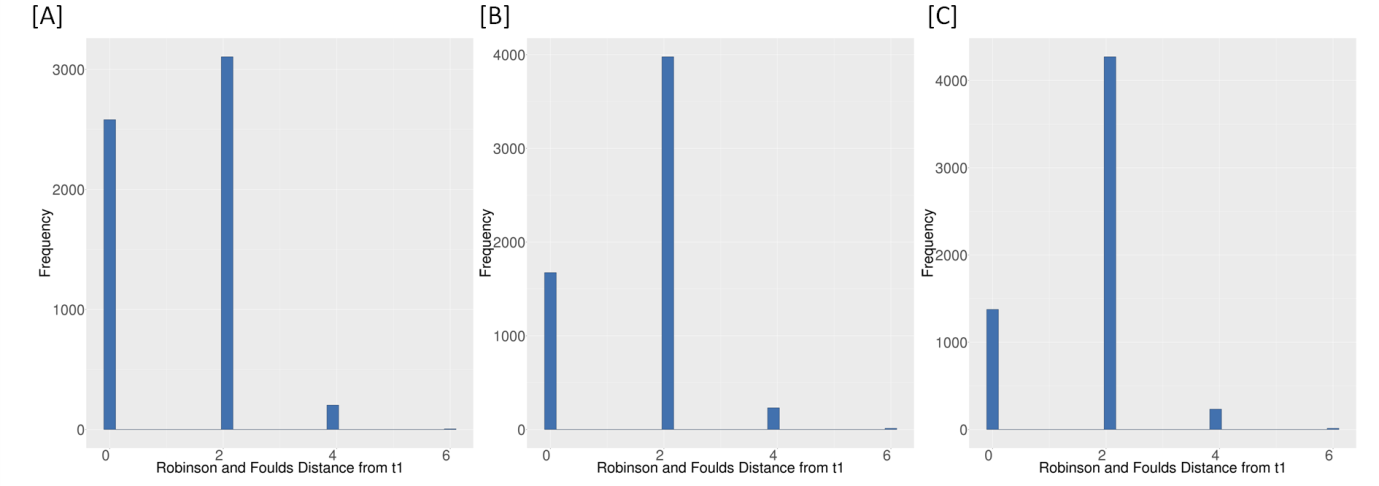


**Supplementary Figure S5: Distribution of Robinson and Foulds distance of individual gene trees from the three topologies t1, t2, and t3 mentioned in the main text. [a] distribution for topology t1 [B] distribution for topology t2 [C] distribution for topology t3.**

**
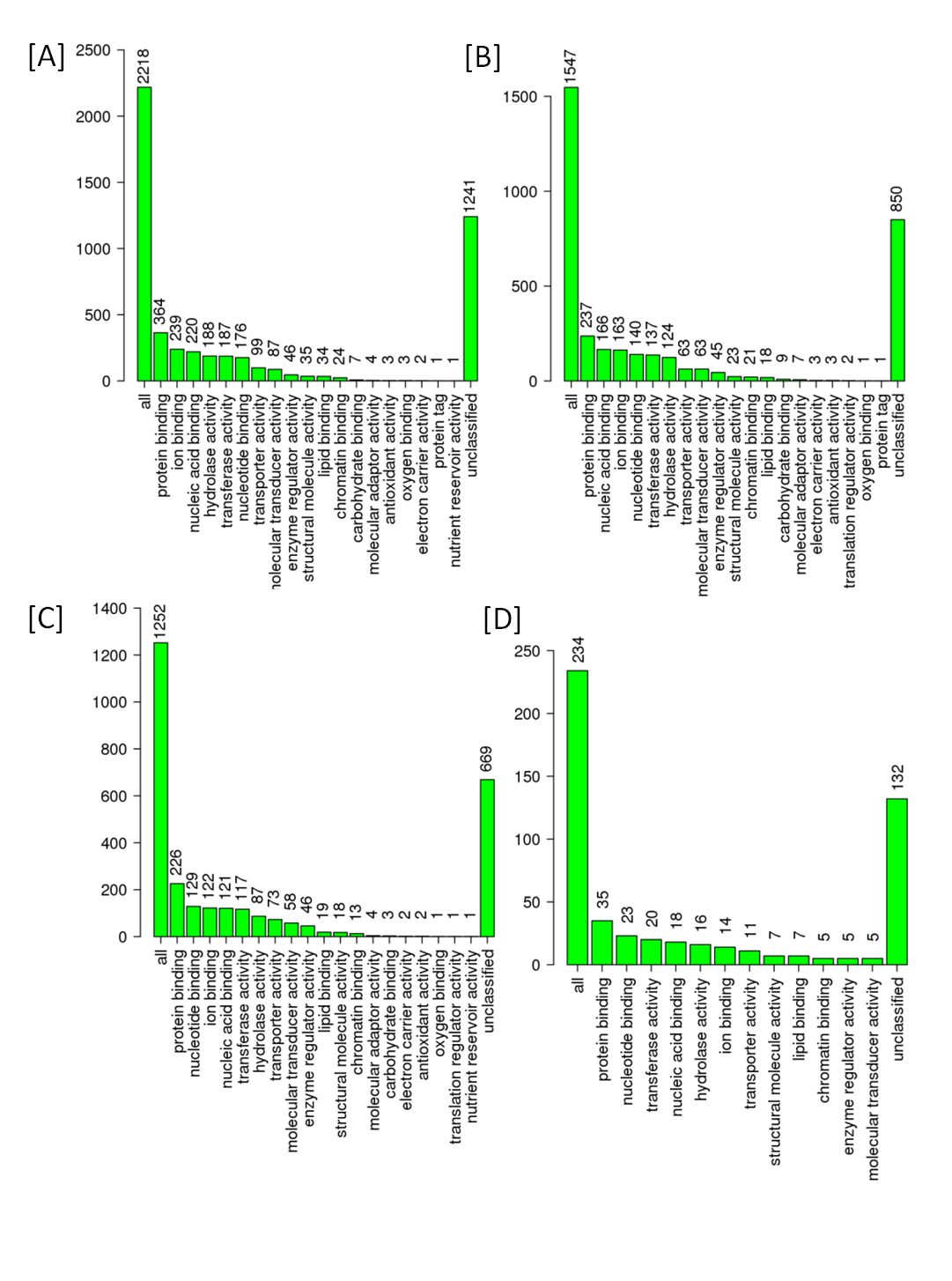
**

**Supplementary Figure S6: Distribution of genes into different molecular function GO categories based on their Robinson and Foulds (RF) distance with topology t1, t2, and t3**

[A] Genes with zero RF distance from t1 [B] Genes with zero RF distance from t2 [C] Genes with zero RF distance from t3 [D] [B] Genes with non-zero RF distance from t1, t2, and t3

**
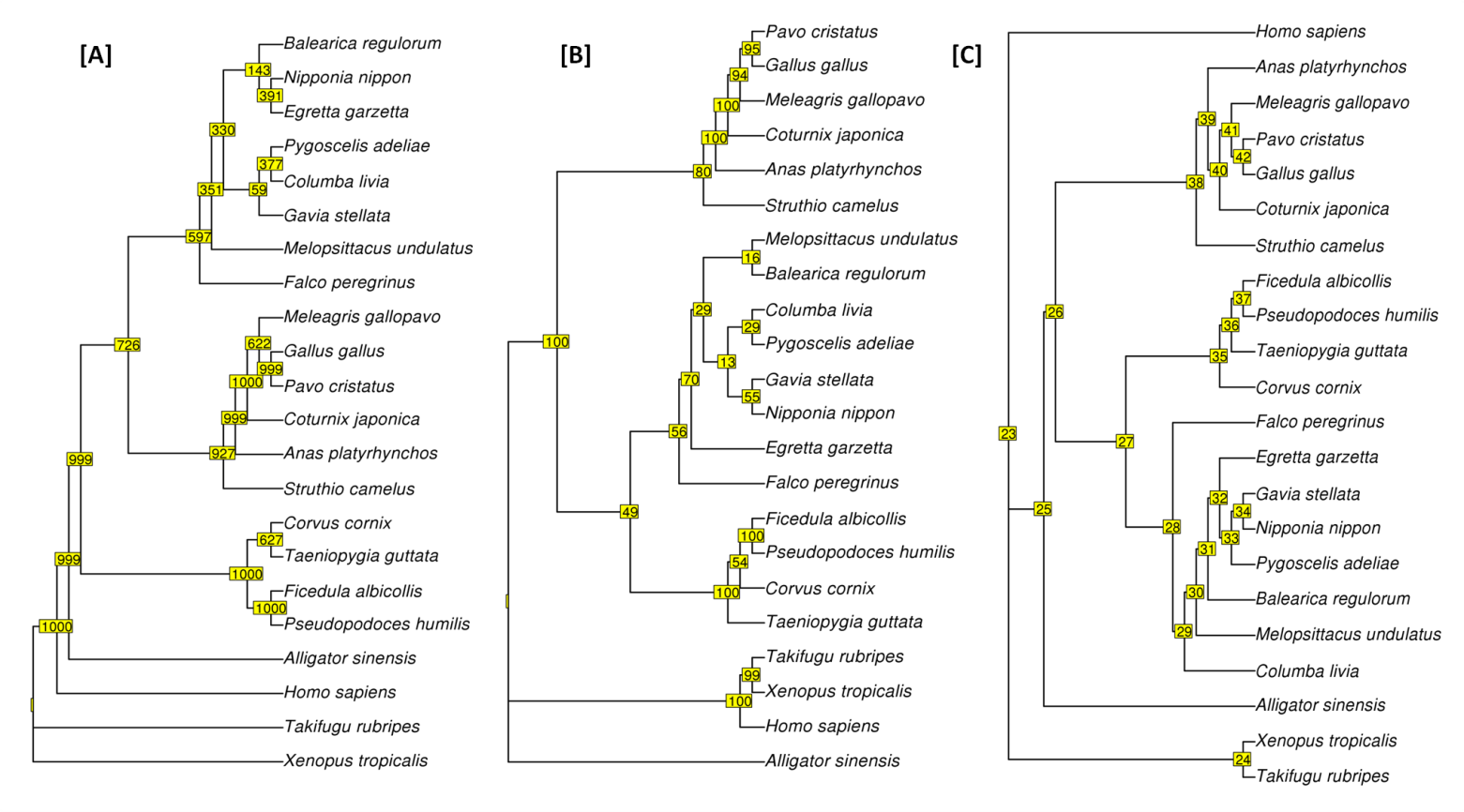
**

**Supplementary Figure S7: Maximum likelihood-based phylogenetic tree of mitochondrial DNA from 22 chordates. The bootstrap values are mentioned in yellow boxes.**

[A] Maximum likelihood phylogeny based on original nucleotide data was constructed using PhyML version 3.1 with the default model of HKY85, [B] Maximum likelihood phylogeny based on the RY-coding (A and G are coded as R and C and T are coded as Y) of the original data was constructed using PhyML version 3.1 with the default model of HKY85, [C] Maximum likelihood phylogeny based on the Binary-coding (A and G are coded as 0 and C and T are coded as 1) of the original data was constructed using RAxML version 8.2.1 with the default model ‘BINGAMMA’ for binary data.

The mitochondrial genome tree showed that peacock is closer to chicken in comparison to turkey in the Galliformes order. The mitochondrial genome sequences of peacock along with and other species from five different classes of Chordates, including Aves (18), Mammalia (1), Reptilia (1), Actinopterygii (1) and Amphibia (1) were used for the construction of the phylogenetic tree.

**
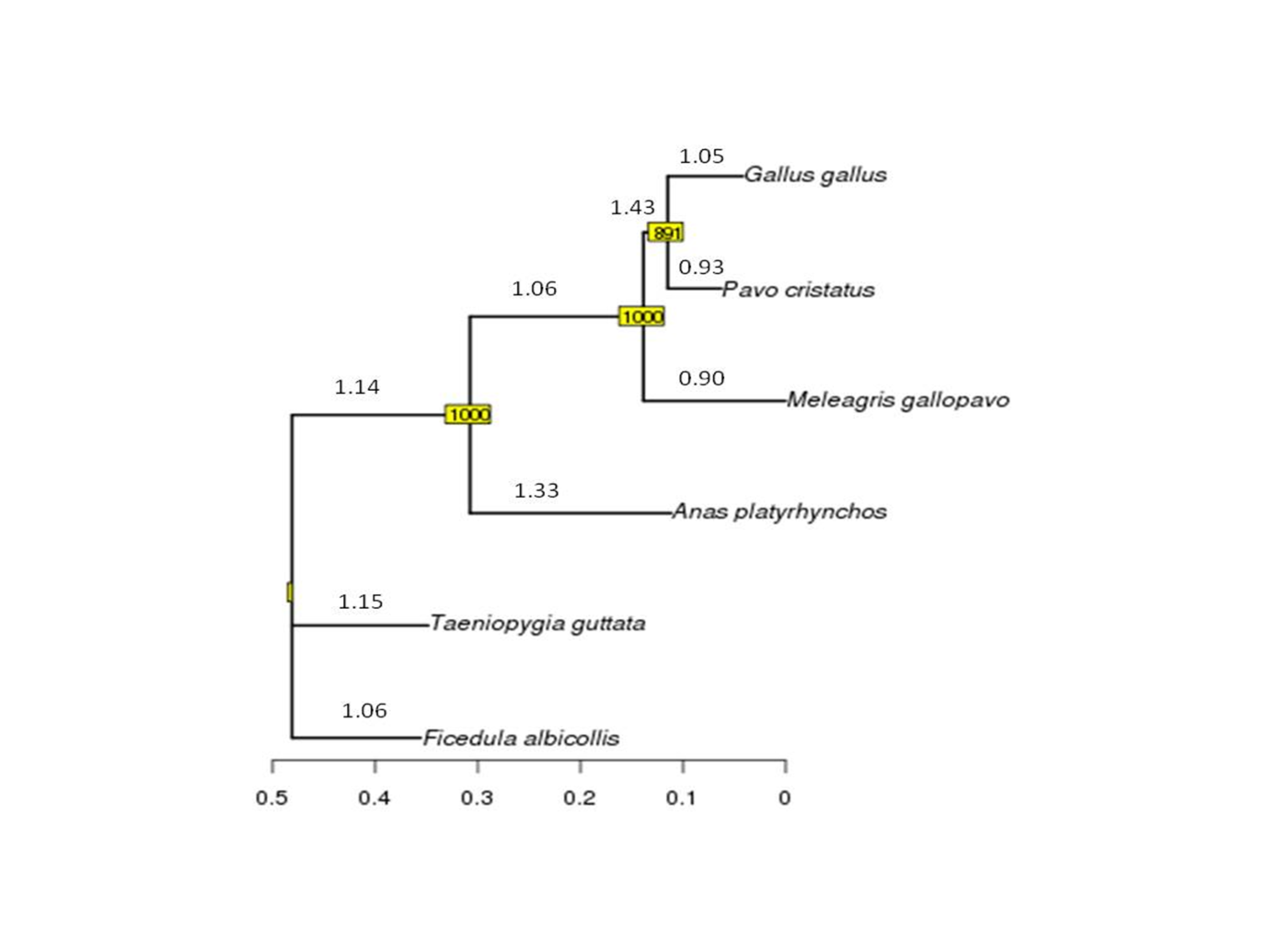
Supplementary Figure S8: Maximum likelihood tree of mitochondrial DNA sequences of the six birds constructed using PhyML.**

The branch-specific ω values are mentioned above the branches, and bootstrap values are mentioned in the Yellow boxes. The tree shows that peacock is closer to chicken than turkey in the Galliformes order. The lower branch-specific ω or dN/dS values for peacock and turkey indicated the slower rate of nucleotide substitutions in their mitochondrial genomes.

Branch length :

(Ficedula_albicollis:0.12557524,Taeniopygia_guttata:0.13286261,(Anas_platyrhynchos:0.19627892,(Meleagris_gallopavo:0.13861580,(Pavo_cristatus:0.05242286,Gallus_gallus:0.07366582)891:0.02367549)1000:0.16902479)1000:0.17353910);

Omega (ω) values :

(Ficedula_albicollis #1.06098 , Taeniopygia_guttata #1.15664 , (Anas_platyrhynchos #1.33944 , (Meleagris_gallopavo #0.904926 , (Pavo_cristatus #0.93322 , Gallus_gallus #1.05377 ) #1.43598 ) #1.06873 ) #1.14805 );

**
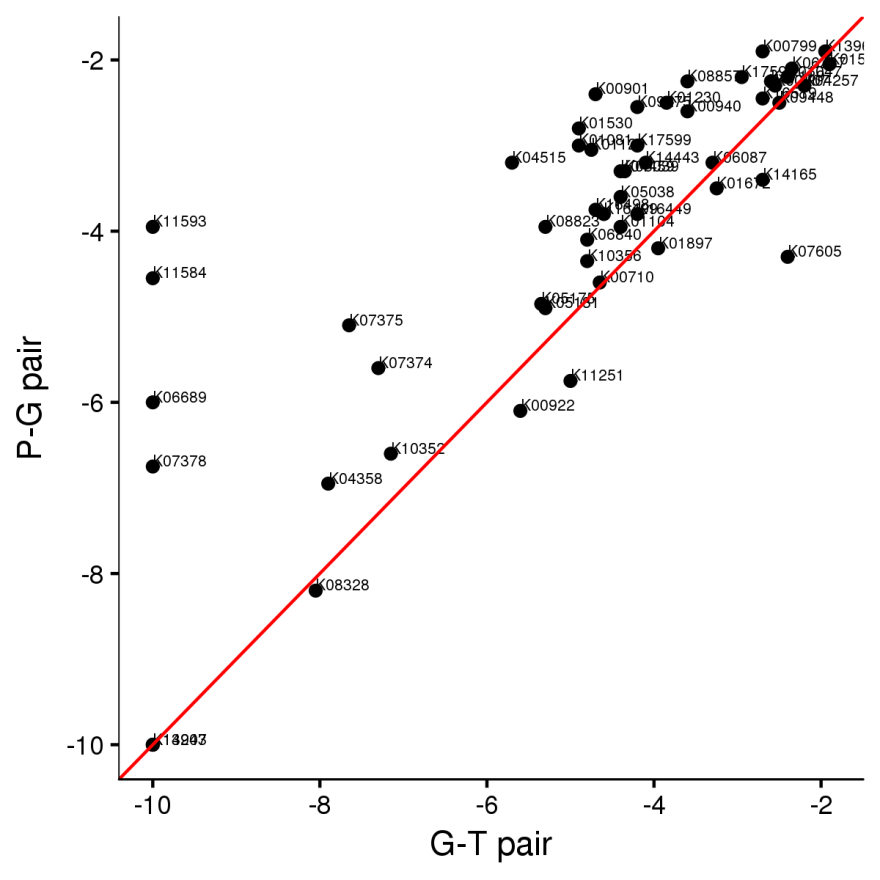
**

**Supplementary Figure S9: The median values of the Ka/Ks ratio for the KEGG categories with ≥ 4 genes were plotted for the peacock-chicken and chicken-turkey pair (Galliformes order).**

The categories such as eukaryotic translation initiation factor 2C (K11593), serine/threonine-protein phosphatase 2A regulatory subunit B (K11584), ubiquitin-conjugating enzyme E2 D/E (K06689), neuroligin (K07378), tubulin beta (K07375) and tubulin alpha (K07374) were found to be rapidly evolving KEGG categories in the peacock genome in comparison to the turkey genome.

**
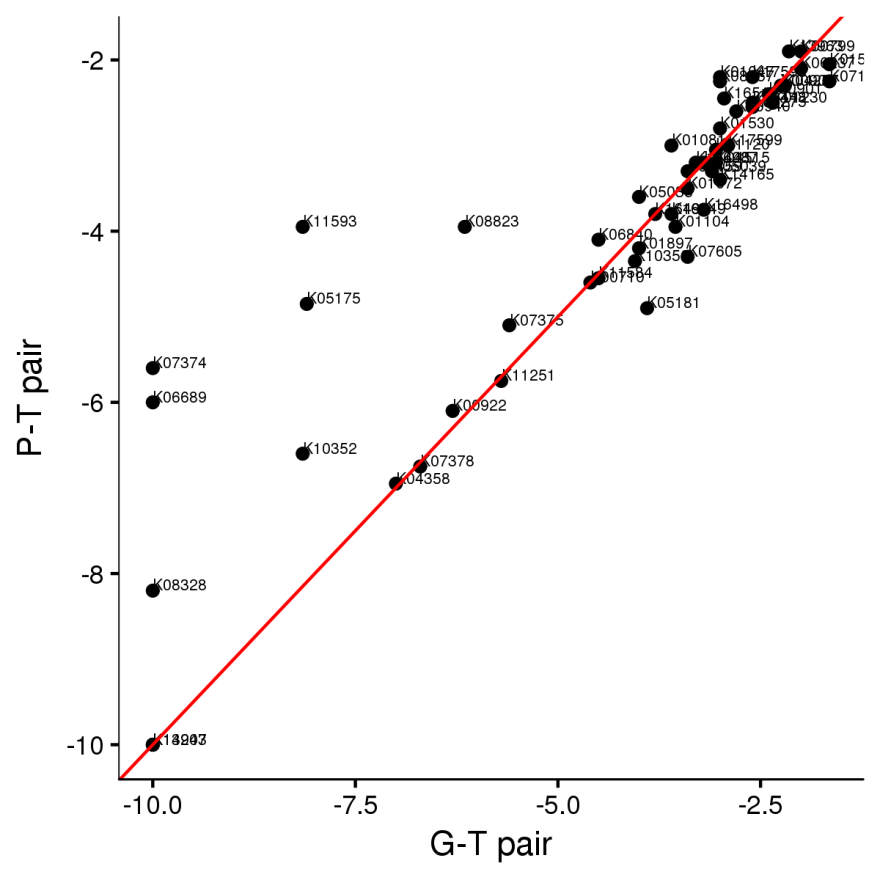
**

**Supplementary Figure S10: The median values of the Ka/Ks ratio for the KEGG categories with ≥ 4 genes were plotted for the peacock-turkey and chicken-turkey pair (Galliformes order).**

The categories such as tubulin alpha (K07374), ubiquitin-conjugating enzyme E2 D/E (K06689), eukaryotic translation initiation factor 2C (K11593), gamma-aminobutyric acid receptor subunit alpha (05175) and guanylate cyclase activator 1 (K08328) were found to be rapidly evolving KEGG categories in the peacock genome in comparison to the chicken genome.

**
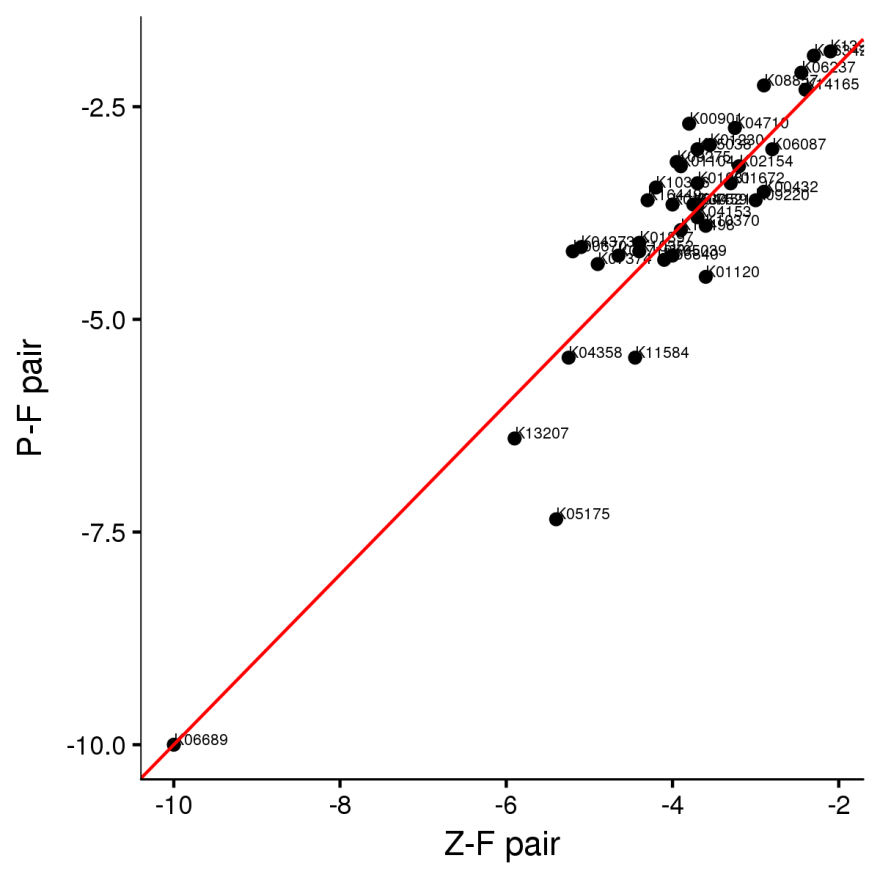
**

**Supplementary Figure S11: The median values of the Ka/Ks ratios for the KEGG categories with ≥ 4 genes were plotted for the peacock-flycatcher and zebra finch-flycatcher pair.**

The KEGG category gamma-aminobutyric acid receptor subunit alpha (K05175) was found to be rapidly evolving in the zebra finch genome in comparison to the peacock genome.

**Supplementary Figure S12: The distribution of different types of orthologs in the six bird genomes and the green anole (out-group).**

**SUPPLEMENTARY TEXT**

**Text S1: Molecular sexing assay**

The CHD1 gene (chromo-helicase-DNA binding protein coding gene) from the genomic DNA was amplified to determine the sex of the peacock using the following pair of universal primers: 2550F – 5’ GTTACTGATTCGTCTACGAGA3’ and 2718R – 5’ATTGAAATGATCCAGTGCTTG3’ ([Ramesh and Mcgowan, 2009](#_ENREF_17)). The PCR was performed using 10 ng of genomic DNA, 0.05 U AmpliTaq Gold polymerase (Life technologies, USA), 200 µM dNTPs, 1X PCR buffer, 1.75 mM MgCl_2_ and 0.5 µM of each forward and reverse primer. Initial denaturation was carried out at 94 °C for 5 minutes followed by a ‘touch-down’ pattern where the denaturation was at 94 °C for 30 seconds, and the annealing temperature was gradually lowered 1 °C per cycle from 65 °C to 50 °C. 30 cycles were run at 50 °C as constant annealing temperature with extension at 72 °C for 40 seconds. A final extension was performed at 72 °C for 5 minutes. PCR product was visualized on 2.5 % Agarose gel by EtBr staining. The molecular sexing experiment will shows a single band (~700 bp) in case of males, which are homogametic (ZZ), and two bands (700 and 500 bp) for females, which are heterogametic (ZW). We observed a single band at ~700 bp (Figure below), which confirmed that it was a male (peacock).


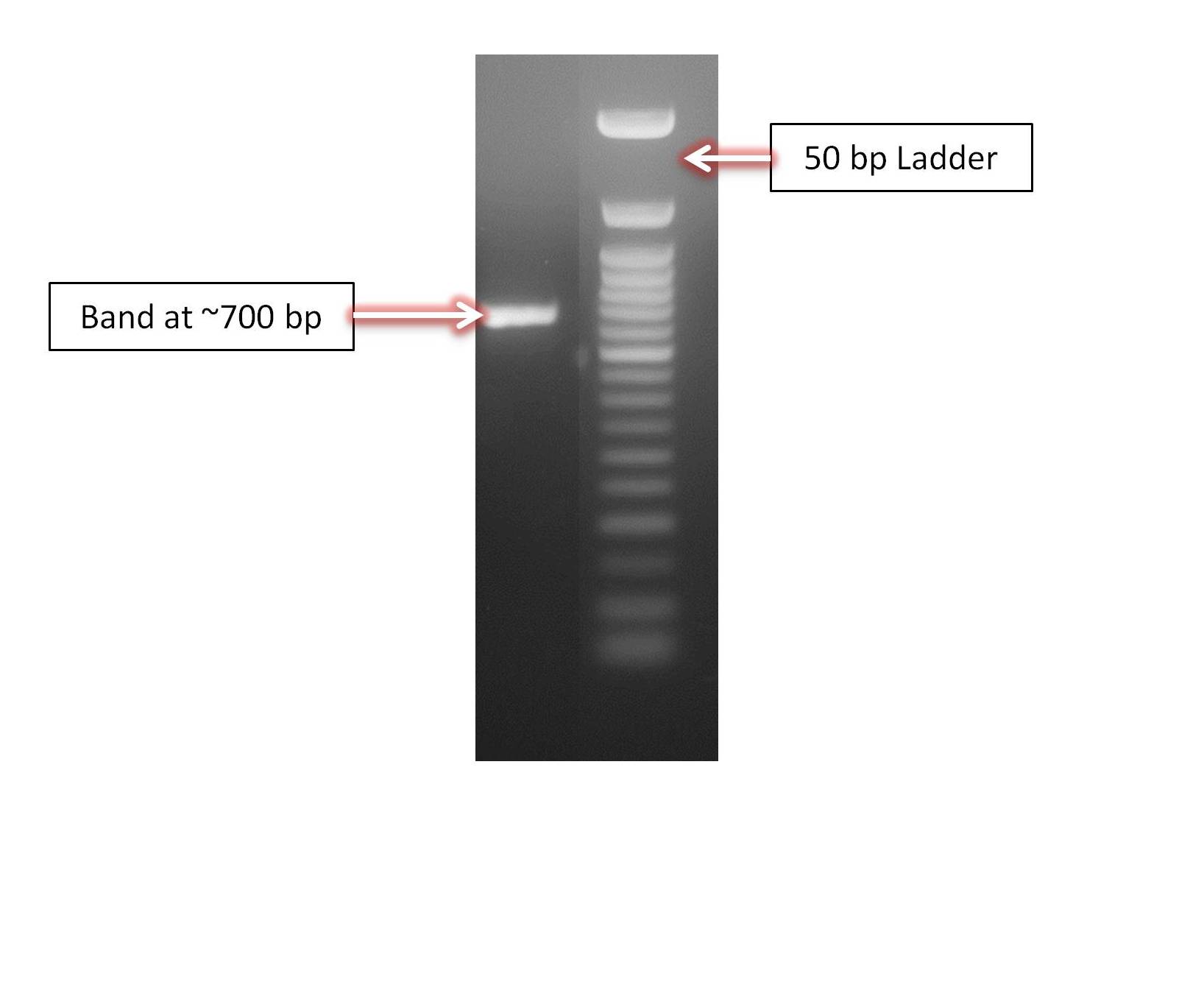


Figure. Molecular sexing of peacock using PCR amplification of CHD1 gene

##

**Text S2: Processing of raw sequence data: Filtering, normalization and error correction**

To minimize the impact of sequencing errors on genomic/contig assembly, the sequence reads were filtered using the following steps.

1. Ambiguous bases (N’s) from 5'-end and 3'-end were trimmed from the sequences using the AmbiguityFiletring.pl script of NGSQCToolkit ([Zahavi, 1975](#_ENREF_20)).

2. Reads with more than five ambiguous bases and length shorter than 80 bp were discarded using the AmbiguityFiletring.pl script of NGSQCToolkit.

3. Reads were further trimmed from the 3'-end using the default parameters if the quality score at this end was less than Q20 using the TrimmingReads.pl script of NGSQCToolkit.

4. Reads containing homopolymers of length greater than 15 bp (>10% of the read length) were trimmed and if the resultant reads were shorter than 80 bp, they were discarded using the HomoPolymerTrimming.pl script of NGSQCToolkit.

5. Final quality check and filtering were performed by extracting only those reads which qualified Q30 score criteria using the IlluQC.pl script of NGSQCToolkit.

## Estimating genome size using the K-mer approach

To estimate the genome size of peacock, a previously used k-mer based approach was followed ([Loyau et al., 2005](#_ENREF_13)). A sequence can be represented as a set of sub-sequences of k nucleotide in length. A read sequence of 'l' bp length should contain (l - k + 1) k-mers if the length of each k-mer is k bp. The k-mer frequencies of each k-mer, calculated from the raw sequence reads, generally follows a Poisson distribution along the sequence depth gradient. Hence, the genome size 'G' can be calculated as G = k_total/k_depth, where k_total is the total number of kmers and k_depth is the sequencing depth of the k-mer with the highest frequency. In this study, K was set to 21.

**Text S3: Genome annotation**

***Repeats Identification***

For the identification of transposable elements and other types of interspersed repeats in the peacock genome, the homology-based search was performed against the Repbase repeat library ([Bao et al., 2015](#_ENREF_4)). The RepeatMasker v4.0.5 (Smit AFA, Hubley R, Green P. 2013. available at http://www.repeatmasker.org) with the NCBI RMBlast v2.2.28 was utilized for this homology search against the aves-specific repeats in the Repbase library.

***tRNA Identification***

tRNAs were predicted from the chromosomes using tRNAscan-SE with its default parameters([Lowe and Eddy, 1997](#_ENREF_12)). A total of 213, 278, 149, and 266 tRNAs were predicted in peacock, chicken, turkey, duck, respectively.

***snoRNA Identification***

Small nucleolar RNAs (snoRNAs) are one of the most abundant groups of ncRNAs in the genome. Their main function is to guide the modification of other ncRNAs, mainly ribosomal RNAs (rRNAs), transfer RNAs (tRNAs) and small nuclear RNAs.

We downloaded snoRNA from human, chicken, turkey, duck, flycatcher, zebra finch, mouse, zebrafish, platypus and lizard from ensemble ([Aken et al., 2016](#_ENREF_2)) and removed the redundancies by clustering at 90% identity. Using the non-redundant snoRNA dataset, BLAST alignment ([Altschul et al., 1990](#_ENREF_3)) was performed against the peacock chromosome using the thresholds of 90%, 90% and 1E-03 for the sequence identity, sequence coverage and E value of the Blast-hits, respectively. This analysis revealed a total of 151 snoRNA in peacock.

***microRNA Identification***

We retrieved 35,828 miRNA sequences from the mirBase database([Griffiths-Jones, 2006](#_ENREF_7)) and clustered them to remove redundancies to form a set of 21,426 miRNA sequences. The non-redundant set was used to identify microRNAs in peacock, chicken, turkey, duck, flycatcher and zebra finch genomes using BLAST with three thresholds: sequence identity > 95%, E-value < 1×10-3, and query coverage > 95%. This procedure identified 540, 852, 446 and 418 microRNAs in peacock, chicken, turkey, duck, flycatcher and zebra finch, respectively.

***Construction of Geneset***

The gene information of chicken, turkey, duck and zebra finch were used to identify putative coding regions of peacock. The gene structure (GFF) and chromosomal/scaffold genomic assemblies of chicken (Gallus gallus), turkey (Meleagris gallopavo), duck (Anas platyrhnchos) and zebra finch (Taeniopygia) were retrieved from Ensembl (Genome browser 86) ([Aken et al., 2016](#_ENREF_2)). For the reconstruction of transcripts, all coding exons along with 150 bp flanks for 25,697 transcripts were extracted from the chicken assembly ([Agaba et al., 2016](#_ENREF_1)).

To construct a comprehensive transcript dataset from the four birds, the chicken transcripts were aligned with the transcripts of turkey, duck and zebra finch to identify the unique transcripts present in the respective genomes. Using the criteria of <50% identity and <30% coverage, 900 unique transcripts from turkey, 2,582 from duck and 4,012 from zebra finch were identified which were not present in chicken. The coding exons of these transcripts along with 150 bp flanking region were also extracted from their respective assemblies. The extracted regions (exon+flanking) from these transcripts were used as the reference for mapping the peacock reads using LASTZ ([Harris, 2007](#_ENREF_8)). To consider a read as mapped, at least 2/3rd of the read length should be present in the matched bases and should have at least 3 matches better than the second best alignment.

LASTZ scoring was as follows:

A C G T
A 11 -33 -20 -39
C -33 16 -30 -20
G -20 -30 16 -33
T -39 -20 -33 11

Gap open penalty = 40, Gap extend penalty =4, Hsp threshold = 300, X drop = 80 and Y drop = 80.

Using the above parameters, a total of 24,029 transcripts were constructed for the peacock genome. Out of these transcripts, 22,122 transcripts were constructed by using chicken transcripts as the template and, 801, 626 and 390 transcripts were constructed using turkey, duck and zebra finch, respectively, as the template.

The reading frame of LASTZ constructed coding gene sequence sequences were validated using corresponding homologous chicken protein sequences. For this validation, the constructed CDS sequences were translated using EMBOSS transeq program and searched against the chicken proteins using BLASTp. The reading frame of a coding gene sequence is considered valid if the best hit showed >80% query and subject coverage with an identity above 80%. Out of peacock coding gene sequences constructed via LASTZ, the reading frame of 21,067 coding gene sequences could be validated using the above-mentioned approach and were used in the subsequent analysis.

AUGUSTUS was used to predict complete gene sequences from the peacock contigs, which predicted 25,963 coding sequences in peacock *de novo* assembly. For the gene prediction, chicken gene sequences were used as the reference and others parameters were kept as default. The predicted CDS were BLAST-aligned against the transcript set constructed using LASTZ with identity ≥50%, e-value < 10^-9^, query and subject coverage ≥ 70%. Out of a total of 30,186 CDS identified by Augustus, 20,189 CDS could match with the transcripts constructed using LASTZ and 90 CDS were confirmed using the transcript datasets of other birds. In addition, a total of 802 complete/partial novel CDS of length ≥ 900 identified using Augustus were also identified. Thus, the final peacock gene set was constructed using the combination of homology and de novo based approach and comprised of 24,831 transcripts and 15,970 genes.

## Text S4: Interspecies variant analysis

To find the interspecies single nucleotide variants (SNVs) between chicken and peacock, all the filtered sequencing reads of peacock were mapped to the chicken genome. The read sequence that aligned to the reference genome were sorted by the start position of their alignment to the reference genome and a consensus sequence was built using BWA(v0.5.9) ([Edgar, 2004](#_ENREF_5)). The difference between chicken and peacock were identified using mpileup, bcftools and Perl scripts provided in SAMtools (v 0.1.19)([Li and Durbin, 2011](#_ENREF_11)) with default parameters. To discard the effects of incorrect read mapping due to mapping of reads on duplicated regions, a cut-off of 50x to 150x was considered for variant calling to remove the variants with too high or too low coverage. As a result, a total of 2,051,161 heterozygous SNVs were identified at a rate of 2.05 SNV per KB. The observed SNV rate in peacock was close to turkey in comparison to the other avian species with respect to chicken genome.

**Text S5:** **Comparison of pairwise ω values**

For the comparison of pairwise ω values, four different pairs were considered: peacock-chicken, peacock-turkey, peacock-flycatcher and peacock-zebra finch. To identify the genes displaying rapid evolution in peacock, the peacock-chicken and peacock-turkey pairs were compared to chicken-turkey, and the peacock-flycatcher and peacock-zebra finch pairs were compared to flycatcher-zebra finch. The orthologous genes used for each of the pairs: peacock-chicken, peacock-turkey and chicken-turkey were 9,271 in number, whereas, the orthologous genes used for the peacock-flycatcher, peacock-zebra finch and flycatcher-zebra finch pairs were 7,727 in number. The orthologous genes were identified using RBH approach mentioned in above text. The functional annotation of these orthologous genes was performed using KEGG database ([Nadachowska-Brzyska et al., 2015](#_ENREF_14)). The median values of the Ka/Ks ratio for the KEGG categories with ≥ 4 genes were plotted for the different pairs and are shown in the following figures.

## Text S6: Orthologous gene clustering

In this study, a gene family is defined as a group of genes descended from a single gene in the most recent common ancestor (TreeFam). The protein sequences from five avian genomes and an out-group green anole were retrieved from ENSEMBL and to this set; the protein sequences of peacock (this study) were added to construct the comprehensive protein dataset to identify the gene families. An all versus all BLASTP was performed on this protein dataset with an E-value <1E-9. For every protein sequence, SOLAR program was used to conjoin the fragment alignments. Similarity (edge) was assigned weight using the Hscore that ranged from 0 to 100. For two genes G1 and G2, the Hscore was calculated using blast bit-score as : score (G1G2) / max (score (G1G1), score (G2G2)). Hierarchical clustering approach using Hcluster_sg was implemented to extract the gene families with the minimum edge density (total number of edges / theoretical number of edges) to be larger than 1/3. The clustering for a gene family would terminate if it already had one or more identified outgroup genes. This resulted in a total of 21,034 gene families in the seven species. The gene families identified in different species are summarized in table and figure below.

**Text S7:** **Comparative genomics: Adaptive sequence divergence in Indian peacock**

To identify the genes possessing nucleotide substitutions responsible for the adaptive evolution and which may provide a selective advantage to the organism, a comparative analysis of the ratio of the rate of non-synonymous to the rate of synonymous substitutions (dN/dS or ω value) was performed. This analysis also helped to measure the relative importance of the genetic drift and selection in causing the nucleotide substitutions. In the pair-wise estimation of dN/dS values, a total of 74 genes showed dN/dS values above one, whereas, 1,429 genes showed higher dN/dS value (4 times) in peacock in comparison to chicken and turkey. The functional analysis of these dN/dS genes with higher dN/dS values revealed that these genes were predominantly involved in different cellular processes such as signal transduction mechanisms, intracellular trafficking, secretion, vesicular transport, post-translational modifications, protein turnover, chaperones, and transcription. The major pathways that showed dN/dS enrichment were MAPK, Wnt, PI3K-Akt, and calcium signaling, cytokine-cytokine receptor interactions, endocytosis, neuroactive ligand-receptor interactions, purine metabolism, and oxidative phosphorylation (Table A provided below).

Furthermore, the identification of the genes showing positive selection in peacock in comparison to chicken, turkey, duck, flycatcher, and zebra finch, was carried out using branch-sites model A implemented in CODEML. All the genes which displayed a fdr q-value <0.1 based on the chi-square test of likelihood scores from the selection model, were considered as the genes under significant positive selection. In total 437 genes showed notable signs of positive selection in peacock. From the functional analysis, it was apparent that these genes belonged to processes such as signal transduction mechanisms, intracellular trafficking, secretion, vesicular transport, post-translational modifications, protein turnover, chaperones, and transcription, cytoskeleton, replication, recombination, and repair. The pathways for genes that showed positive selection were Ras, MAPK, PI3K-Akt, and Wnt signalling, purine metabolism, axon guidance, neuroactive ligand-receptor interaction, pyrimidine metabolism, glycolysis/gluconeogenesis, and complement and coagulation cascade (Table B provided below).

The same set of 5,907 combined orthologs was also utilized for the unique substitution analysis. In this analysis gene displaying the unique amino acid substitutions in peacock in comparison to all the other bird genomes, were identified using custom-made Perl scripts. A total of 3,237 peacock coding gene sequences showed unique substitutions, out of which 116 genes showed unique substitutions which significantly affect the function of the protein based on SIFT analysis. These genes with functionally critical unique substitutions belonged to different cellular processes such as signal transduction mechanisms, intracellular trafficking, secretion, vesicular transport, post-translational modifications, protein turnover, chaperones, replication, recombination, repair, and amino acid metabolism and transport. The genes were predominantly involved in the pathways: complementation and coagulation cascade, protein processing in endoplasmic reticulum, axon guidance, endocytosis, ras signaling, arginine and proline metabolism, and pyrimidine metabolism.

Table A. GO enrichemnt of the genes dispalying msa in peacock (99 in munber). Only GO caytegories with p-value < 0.1 are displayed

| GO ID | Description | P Value | FDR q-value |
| --- | --- | --- | --- |
| GO:0051540 | metal cluster binding | 0.001928015 | 0.142673109 |
| GO:0005539 | glycosaminoglycan binding | 0.044742061 | 0.835717084 |
| GO:0098772 | molecular function regulator | 0.045001701 | 0.835717084 |
| GO:0008233 | peptidase activity | 0.047182071 | 0.835717084 |
| GO:1901681 | sulfur compound binding | 0.079795738 | 0.835717084 |
| GO:0009055 | electron carrier activity | 0.094099523 | 0.835717084 |

Table B. GO enrichemnt of the genes dispalying positive selection in peacock (437 in munber). Only GO caytegories with p-value < 0.1 are displayed

| GO ID | Description | P Value | FDR q-value |
| --- | --- | --- | --- |
| GO:0051540 | metal cluster binding | 0.006428 | 0.293353 |
| GO:0046983 | protein dimerization activity | 0.007928 | 0.293353 |
| GO:0016817 | hydrolase activity, acting on acid anhydrides | 0.012608 | 0.310988 |
| GO:0030554 | adenyl nucleotide binding | 0.018728 | 0.319832 |
| GO:0005539 | glycosaminoglycan binding | 0.02161 | 0.319832 |
| GO:0019838 | growth factor binding | 0.026211 | 0.323272 |
| GO:0000287 | magnesium ion binding | 0.037268 | 0.369411 |
| GO:0005509 | calcium ion binding | 0.039936 | 0.369411 |
| GO:0019899 | enzyme binding | 0.048305 | 0.397174 |
| GO:0042802 | identical protein binding | 0.073068 | 0.507304 |
| GO:1901681 | sulfur compound binding | 0.07541 | 0.507304 |

Table C. KEGG classification of the genes dispalying unique substitutions

| **Pathway** | **Number of Unique Substituions** |
| --- | --- |
| **Neuroactive ligand-receptor interaction** | 48 |
| **MAPK signaling pathway** | 40 |
| **Protein processing in endoplasmic reticulum** | 38 |
| **Purine metabolism** | 32 |
| **Calcium signaling pathway** | 30 |
| **R0 transport** | 25 |
| **Lysosome** | 23 |
| **N-Glycan biosynthesis** | 19 |
| **Ribosome biogenesis in eukaryotes** | 19 |
| **Ras signaling pathway** | 19 |
| **Endocytosis** | 19 |
| **PI3K-Akt signaling pathway** | 19 |
| **Cytokine-cytokine receptor interaction** | 18 |
| **Cell adhesion molecules (CAMs)** | 18 |
| **Glycerophospholipid metabolism** | 17 |
| **Aminoacyl-tR0 biosynthesis** | 17 |
| **Axon guidance** | 17 |
| **Cell cycle** | 16 |
| **Glycolysis / Gluconeogenesis** | 15 |
| **ABC transporters** | 15 |
| **R0 degradation** | 15 |
| **Ubiquitin mediated proteolysis** | 15 |
| **Oxidative phosphorylation** | 14 |
| **FoxO signaling pathway** | 14 |
| **Glycerolipid metabolism** | 13 |
| **Sphingolipid metabolism** | 13 |
| **Rap1 signaling pathway** | 13 |
| **Complement and coagulation cascades** | 13 |
| **Mucin type O-Glycan biosynthesis** | 12 |
| **Inositol phosphate metabolism** | 12 |
| **Peroxisome** | 12 |
| **D0 replication** | 11 |
| **Spliceosome** | 11 |
| **NF-kappa B signaling pathway** | 11 |
| **Valine, leucine and isoleucine degradation** | 10 |
| **Lysine degradation** | 10 |
| **Homologous recombination** | 10 |
| **p53 signaling pathway** | 10 |
| **Phagosome** | 10 |
| **Wnt signaling pathway** | 10 |

Table D. EggNOG classification of the genes dispalying signs of adaptive sequence divergence.

PS-positive selection, PSS- having positively selected amino acids, US-Unique substitutions, SIFT-having substitution with functional impact, MSA-multiple signs of adaptive evolution (more than two of: higher pair-wise dN/dS, positive selection, unique substituion)

| eggNOG category | Description | dN/dS >= 4X | dN/dS > 1 | PS | PSS | US | SIFT | MSA |
| --- | --- | --- | --- | --- | --- | --- | --- | --- |
| A | RNA processing and modification | 23 | 1 | 9 | 6 | 43 | 0 | 0 |
| B | Chromatin structure and dynamics | 16 | 0 | 4 | 2 | 26 | 2 | 2 |
| C | Energy production and conversion | 20 | 2 | 9 | 10 | 65 | 2 | 2 |
| D | Cell cycle control, cell division, chromosome partitioning | 15 | 0 | 9 | 5 | 46 | 2 | 2 |
| E | Amino acid transport and metabolism | 22 | 1 | 12 | 11 | 85 | 9 | 8 |
| F | Nucleotide transport and metabolism | 9 | 0 | 7 | 8 | 29 | 1 | 1 |
| G | Carbohydrate transport and metabolism | 29 | 2 | 8 | 9 | 103 | 4 | 2 |
| H | Coenzyme transport and metabolism | 5 | 0 | 4 | 2 | 21 | 1 | 1 |
| I | Lipid transport and metabolism | 22 | 0 | 14 | 14 | 101 | 5 | 3 |
| J | Translation, ribosomal structure and biogenesis | 40 | 1 | 16 | 12 | 88 | 2 | 2 |
| K | Transcription | 100 | 3 | 30 | 28 | 184 | 4 | 4 |
| L | Replication, recombination and repair | 18 | 0 | 17 | 14 | 101 | 7 | 7 |
| M | Cell wall/membrane/envelope biogenesis | 3 | 1 | 4 | 4 | 10 | 0 | 0 |
| N | Cell motility | 1 | 0 | 0 | 0 | 4 | 0 | 0 |
| O | Posttranslational modification, protein turnover, chaperones | 110 | 4 | 57 | 47 | 321 | 12 | 10 |
| P | Inorganic ion transport and metabolism | 34 | 1 | 13 | 12 | 87 | 3 | 3 |
| Q | Secondary metabolites biosynthesis, transport and catabolism | 5 | 0 | 3 | 3 | 40 | 0 | 0 |
| R | General function prediction only | 250 | 14 | 81 | 64 | 555 | 22 | 17 |
| S | Function unknown | 317 | 25 | 72 | 59 | 543 | 18 | 18 |
| T | Signal transduction mechanisms | 167 | 3 | 69 | 51 | 390 | 15 | 12 |
| U | Intracellular trafficking, secretion, and vesicular transport | 122 | 0 | 46 | 46 | 351 | 10 | 8 |
| V | Defense mechanisms | 3 | 0 | 5 | 3 | 12 | 0 | 0 |
| W | Extracellular structures | 21 | 1 | 1 | 1 | 19 | 2 | 1 |
| Y | Nuclear structure | 0 | 0 | 0 | 0 | 5 | 0 | 0 |
| Z | Cytoskeleton | 55 | 3 | 18 | 14 | 94 | 3 | 3 |

**Text S8:** **Evolution of Ligand/Receptors/Regulators and Effectors genes in pathways**

In several signaling pathways, it was observed that the extracellular ligand and regulators, receptors, and in some cases the final effectors showed adaptive evolution. In the case of wnt signaling pathway, the inhibitors such as WIF1 and DKK, and final effector such as PPARD showed adaptive evolution (Figure is shown below).

In the case of Jak-Stat pathway only, the receptor and final effector Bcl-XL showed adaptive divergence

*Link for the pathway:* http://www.kegg.jp/kegg-bin/show_pathway?@ko04630/reference%3dwhite/default%3d%23bfffbf/K05081/K04570/K04456

For the cAMP signaling pathway, the GPCR receptor and effectors GLI3 and Akt displayed signs of adaptive evolution

*Link for the pathway:* http://www.kegg.jp/kegg-bin/show_pathway?@ko04024/reference%3dwhite/default%3d%23bfffbf/K04197/K04948/K04456/K06230/K01540

Additionally, for the Ras and Rap1 signaling the GF ligand, RTK receptor, a few final effectors such as Bcl-X, PAR6, Akt, and TSP1 showed adaptive divergence in peacock

*Link for the pathway:* http://www.kegg.jp/kegg-bin/show_pathway?@ko04015/reference%3dwhite/default%3d%23bfffbf/K12362/K02582/K05461/K05090/K05096/K05097/K16857/K05692/K06093/K04456,

*Link for the pathway:* http://www.kegg.jp/kegg-bin/show_pathway?@ko04014/reference%3dwhite/default%3d%23bfffbf/K02582/K05461/K05090/K05096/K05097/K12362/K04456/K04570/K17635/K07889


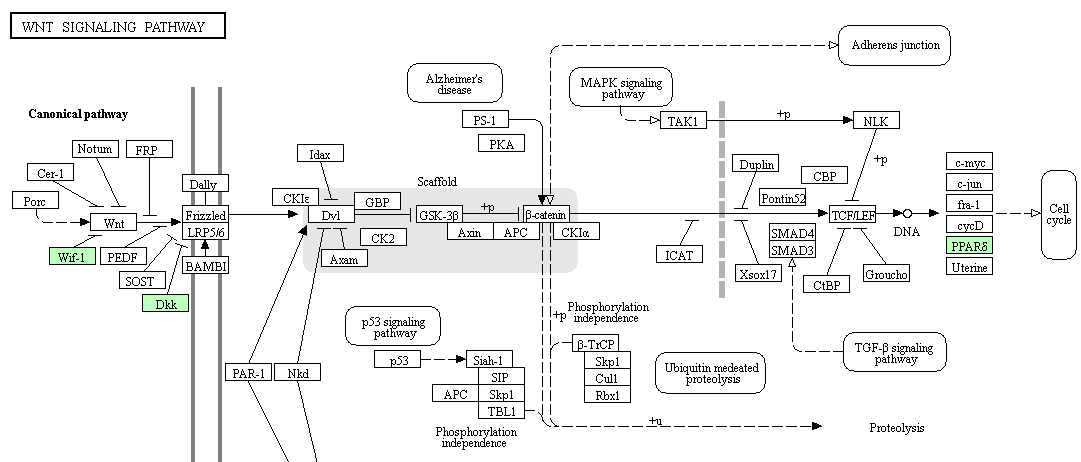


Figure: Genes of wnt signaling pathway showing adaptive evolution are highlighted in green.

For other pathways, please copy and paste the link on the web browser to retrieve the pathway with highlighted genes on KEGG reference pathway

**Text S9:** **Interacting protein pairs which show signs of sequence divergence in peacock**

The interacting protein pairs for chicken were downloaded from the STRING database ([Nadachowska‐Brzyska et al., 2016](#_ENREF_15)). Among all the interacting pairs, the proteins showing similar signs of sequence divergence and adaptive evolution in peacock were filtered and considered to be co-evolving. The numbers of interacting gene-pairs showing co-evolution in terms of divergence in the coding gene sequences are shown in the following table.

| Divergence | Number of gene-pairs |
| --- | --- |
| Positive selection and unique substitutions | 64 |
| Positive selection | 112 |
| Positively selected amino acid sites | 96 |
| dN/dS > 4x | 314 |
| Unique substitutions | 4343 |

**Text S10:** **Identification of feather-related genes**

To construct a reference database of feather and genes related to the feather development process, the term ‘feather’ was searched in NCBI nucleotide database and mRNA sequences were retrieved. The annotations of these mRNA sequences were manually reviewed to remove the ambiguous hits. The feather and related genes were also retrieved from Ensembl database from five avian species including chicken, duck, flycatcher, turkey and zebra finch. In addition, the gene family PTHR31203, which contains feather keratin genes from multiple species, was retrieved from Ensembl database.

The feather genes from all sources were combined to form a single gene set, and the redundancies were removed using CDHIT to create the reference dataset of 2,146 genes. The reference feather gene dataset were aligned by BLASTN (e < 1E-10) against the genomes of six birds including peacock, chicken, duck, flycatcher, turkey and zebra finch. The reference feather dataset was aligned against the CDS datasets of each of the six birds to identify the homologous CDS sequences.

**Text S11: Additional immune system regulators with adaptive sequence divergence in peacock**

The other regulators of immune response also showed multiple signs of adaptation in peacock. The KIT ligand gene, which is involved in hematopoiesis, mast cell development, cellular migration and melanogenesis via PI3K-AkT signaling and MAPK signaling pathway, showed unique substitutions and positive selection with positively selected sites. The GATA-6 gene, which is a transcription factor involved in the protection of gastric epithelium from bacterial infections, showed positive selection with positively selected sites and a unique substitution. Another transcription factor gene, Interferon regulatory factor 8 (IRF8), which is involved in IFN-alpha and IFN beta-mediated signaling upon viral infection, showed multiple unique substitutions. The TNFSF15 gene coding for TNF family cytokine, which causes angiogenesis inhibition by reducing the endothelial cell proliferation, showed positive selection with positively selected sites, and unique substitutions in peacock. The IFRD1 gene, which is involved in regulating the neutrophil activity, showed unique substitutions.

**Text S12: MSA genes involved in Body Dimensions related phenotypes**

Follicle stimulating hormone receptor (FSHR), which is involved in regulating the cell growth, differentiation, and body dimensions of birds via cAMP-mediated PI3K-AKT and SRC-ERK1/2 signaling ([Fayeye et al., 2006](#_ENREF_6)), showed multiple unique substitutions. Several genes such as MMP2, BMP7, TRAF6, TNF3, Neurochondrin, IGF, and NOX4, regulating bone morphogenesis and development in birds showed divergence as well as adaptive evolution in peacock. These genes primarily function as ligands or receptors for Wnt-beta-catenin, TGF-beta, p70S6K and PEDF signaling pathways. From these observations, it appears that the adaptive evolution of intracellular signaling and early development genes, which play significant roles in bone and skeletal muscle development, are perhaps beneficial for supporting its body dimensions.

**Text S13: MSA genes involved in other cellular processes**

Among the other genes that displayed multiple signs of adaptation, BRCA2, DNA-PKcs, FANCC, and INO80 genes were involved in the DNA double-strand break repair and recombination, FBXO15, USP53, and PSMD1-26S were part of ubiquitin-proteasomal protein degradation system, HERPUD1 and HSP90B1 genes were involved in stress response, and METTL5 gene had protein methyltransferase activity. Thus, DNA repair and protein turnover and modification were among the other cellular processes where a notable number of genes showed MSA.

# ABBREVIATIONS

MAPK Mitogen-Activated Protein Kinase

Jak-STAT Janus kinase/signal transducers and activators of transcription

Wnt Wingless-type MMTV integration site family member

TLR Toll-like receptor

TGF-β Transforming growth factor beta

FGF Fibroblast growth factors

BMP Bone morphogenetic proteins

cAMP Cyclic adenosine monophosphate

GPCR G-protein-coupled receptors

TGFBR3 Transforming Growth Factor Beta Receptor 3

TGFBRAP1 Transforming Growth Factor Beta Receptor Associated Protein 1

TAB3 TGF-Beta Activated Kinase 1/MAP3K7 Binding Protein 3

WIF1 WNT Inhibitory Factor 1

DKK2 Dickkopf WNT Signaling Pathway Inhibitor 2

BRK-3 Breast Tumor Kinase-3

CRIM1 Cysteine Rich Transmembrane BMP Regulator 1

Notch-2 Neurogenic locus notch homolog protein 2

C5 Complement Component 5

C8 Complement Component 8

MAC membrane attack complex

CSF-1R Colony Stimulating Factor 1 Receptor

NF-ĸB nuclear factor kappa-light-chain-enhancer of activated B cells

MYD88 Myeloid Differentiation Primary Response 88

TRADD Tumor necrosis factor receptor type 1-associated DEATH domain protein

SIGIRR Single Ig IL-1-related receptor

MAP3K14 Mitogen-Activated Protein Kinase Kinase Kinase 14

NLRC3 NLR Family CARD Domain Containing 3

ITGAV Integrin, Alpha V

AQP3 Aquaporin 3

SDC4 Syndecan 4

FLT4 Fms-related tyrosine kinase 4

NFATC3 Nuclear Factor Of Activated T-Cells 3

IL12B Interleukin 12B

CTLA4 Cytotoxic T-Lymphocyte Associated Protein 4

SSC4D Scavenger Receptor Cysteine Rich Family Member With 4 Domains

MMP2 matrix metalloproteinase-2

TRAF6 TNF Receptor Associated Factor 6

TNF Tumor Necrosis Factor

IGF Insulin-like growth factor

NOX4 NADPH Oxidase 4

p70S6K Ribosomal protein S6 kinase beta-1

PEDF Pigment epithelium-derived factor

BRCA2 Breast And Ovarian Cancer Susceptibility Protein 2

DNA-PKcs DNA-dependent protein kinase, catalytic subunit

FANCC Fanconi Anemia Complementation Group C

FBXO15 F-Box Protein 15

USP53 Ubiquitin Specific Peptidase 53

HERPUD1 Homocysteine Inducible ER Protein With Ubiquitin Like Domain 1

HSP90B1 Heat Shock Protein 90 Beta Family Member 1

METTL5 Methyltransferase Like 5

# REFERENCES

Agaba, M., Ishengoma, E., Miller, W.C., Mcgrath, B.C., Hudson, C.N., Bedoya Reina, O.C., Ratan, A., Burhans, R., Chikhi, R., Medvedev, P., Praul, C.A., Wu-Cavener, L., Wood, B., Robertson, H., Penfold, L., and Cavener, D.R. (2016). Giraffe genome sequence reveals clues to its unique morphology and physiology. *Nat Commun* 7**,** 11519.

Aken, B.L., Ayling, S., Barrell, D., Clarke, L., Curwen, V., Fairley, S., Fernandez Banet, J., Billis, K., Garcia Giron, C., Hourlier, T., Howe, K., Kahari, A., Kokocinski, F., Martin, F.J., Murphy, D.N., Nag, R., Ruffier, M., Schuster, M., Tang, Y.A., Vogel, J.H., White, S., Zadissa, A., Flicek, P., and Searle, S.M. (2016). The Ensembl gene annotation system. *Database (Oxford)* 2016.

Altschul, S.F., Gish, W., Miller, W., Myers, E.W., and Lipman, D.J. (1990). Basic local alignment search tool. *J Mol Biol* 215**,** 403-410.

Bao, W., Kojima, K.K., and Kohany, O. (2015). Repbase Update, a database of repetitive elements in eukaryotic genomes. *Mob DNA* 6**,** 11.

Edgar, R.C. (2004). MUSCLE: multiple sequence alignment with high accuracy and high throughput. *Nucleic Acids Res* 32**,** 1792-1797.

Fayeye, T., Ayorinde, K., Ojo, V., and Adesina, O. (2006). Frequency and influence of some major genes on body weight and body size parameters of Nigerian local chickens. *Livestock research for rural development* 18**,** 37.

Griffiths-Jones, S. (2006). miRBase: the microRNA sequence database. *Methods Mol Biol* 342**,** 129-138.

Harris, R.S. (2007). *Improved pairwise alignment of genomic dna.* Pennsylvania State University.

Huxley, T.H. (1968). *On the origin of species.* University of Michigan P.

Lee, C.-Y., Hsieh, P.-H., Chiang, L.-M., Chattopadhyay, A., Li, K.-Y., Lee, Y.-F., Lu, T.-P., Lai, L.-C., Lin, E.-C., and Lee, H. (2018). Whole-genome de novo sequencing reveals unique genes that contributed to the adaptive evolution of the Mikado pheasant. *Gigascience* 7**,** giy044.

Li, H., and Durbin, R. (2011). Inference of human population history from individual whole-genome sequences. *Nature* 475**,** 493-496.

Lowe, T.M., and Eddy, S.R. (1997). tRNAscan-SE: a program for improved detection of transfer RNA genes in genomic sequence. *Nucleic Acids Res* 25**,** 955-964.

Loyau, A., Jalme, M.S., and Sorci, G. (2005). Intra- and Intersexual Selection for Multiple Traits in the Peacock (Pavo cristatus). *Ethology* 111**,** 810-820.

Nadachowska-Brzyska, K., Li, C., Smeds, L., Zhang, G., and Ellegren, H. (2015). Temporal dynamics of avian populations during Pleistocene revealed by whole-genome sequences. *Current Biology* 25**,** 1375-1380.

Nadachowska‐Brzyska, K., Burri, R., Smeds, L., and Ellegren, H. (2016). PSMC analysis of effective population sizes in molecular ecology and its application to black‐and‐white Ficedula flycatchers. *Molecular Ecology* 25**,** 1058-1072.

Ouyang, Y.-N., Yang, Z.-Y., Li, D.-L., Huo, J.-L., Qian, K., and Miao, Y.-W. (2009). Genetic Divergence between Pavo muticus and Pavo cristatus by Cyt b Gene. *Journal of Yunnan Agricultural University* 2**,** 014.

Ramesh, K., and Mcgowan, P. (2009). On the current status of Indian peafowl Pavo cristatus (Aves: Galliformes: Phasianidae): keeping the common species common. *Journal of Threatened Taxa* 1**,** 106-108.

Tiley, G., Kimball, R., Braun, E., and Burleigh, J. (2018). Comparison of the Chinese bamboo partridge and red Junglefowl genome sequences highlights the importance of demography in genome evolution. *BMC genomics* 19**,** 336.

Wu, Y., Zhang, Y., Hou, Z., Fan, G., Pi, J., Sun, S., Chen, J., Liu, H., Du, X., and Shen, J. (2018). Population genomic data reveal genes related to important traits of quail. *Gigascience* 7**,** giy049.

Zahavi, A. (1975). Mate selection—A selection for a handicap. *Journal of Theoretical Biology* 53**,** 205-214.

Zhang, G., Li, B., Li, C., Gilbert, M.T.P., Jarvis, E.D., and Wang, J. (2014). Comparative genomic data of the Avian Phylogenomics Project. *GigaScience* 3**,** 26.
